# HOOK1 Inhibits the Progression of Renal Cell Carcinoma via TGF‐*β* and TNFSF13B/VEGF‐A Axis

**DOI:** 10.1002/advs.202206955

**Published:** 2023-04-21

**Authors:** Lei Yin, Wenjia Li, Xuxiao Chen, Ronghao Wang, Tao Zhang, Jialin Meng, Zhao Li, Li Xu, Rui Yin, Bo Cheng, Huan Yang

**Affiliations:** ^1^ Department of Urology Putuo People's Hospital Tongji University Shanghai 200060 P. R. China; ^2^ Department of Urology Ruijin Hospital Shanghai Jiao Tong University School of Medicine Shanghai 200025 P. R. China; ^3^ Department of Cardiovascular Medicine Ruijin Hospital Shanghai Jiao Tong University School of Medicine Shanghai 200025 P. R. China; ^4^ Department of General Surgery Hepatobiliary Surgery Shanghai Institute of Digestive Surgery Ruijin Hospital Shanghai Jiao Tong University School of Medicine Shanghai 200025 P. R. China; ^5^ Department of Biochemistry and Molecular Biology School of Basic Medical Sciences Southwest Medical University Luzhou 646000 P. R. China; ^6^ Department of Urology The First Affiliated Hospital of Anhui Medical University Anhui Province Key Laboratory of Genitourinary Diseases Anhui Medical University Hefei 230032 P. R. China; ^7^ Department of Anesthesiology Xiangya Hospital Central South University Changsha 410008 P. R. China; ^8^ Department of Anesthesiology The First People's Hospital of Changde Changde 415000 P. R. China; ^9^ Center for Reproductive Medicine Shandong University Jinan 250012 P. R. China; ^10^ Department of Urology The Affiliated Hospital of Southwest Medical University Luzhou 646000 P. R. China; ^11^ Department of Urology Tongji Hospital Tongji Medical College of Huazhong University of Science and Technology Wuhan 430030 P. R. China

**Keywords:** HOOK1, renal cell carcinoma, TGF‐Î^2^ signaling, TNFSF13B, tumor metastasis

## Abstract

Accumulating evidence shows HOOK1 disordered in human malignancies. However, the clinicopathological and biological significance of HOOK1 in renal cell carcinoma (RCC) remains rarely studied. In this study, the authors demonstrate that HOOK1 is downregulated in RCC samples with predicted poorer clinical prognosis. Mechanistically, HOOK1 inhibits tumor growth and metastasis via canonical TGF‐*β*/ALK5/p‐Smad3 and non‐canonical TGF‐*β*/MEK/ERK/c‐Myc pathway. At the same time, HOOK1 inhibits RCC angiogenesis and sunitinib resistance by promoting degradation of TNFSF13B through the ubiquitin‐proteasome pathway. In addition, HOOK1 is transcriptionally regulated by nuclear factor E2F3 in VHL dependent manner. Notably, an agonist of HOOK1, meletin, is screened and it shows antitumor activity more effectively when combined with sunitinib or nivolumab than it is used alone. The findings reveal a pivotal role of HOOK1 in anti‐cancer treatment, and identify a novel therapeutic strategy for renal cell carcinoma.

## Graphical abstract

Metastasis is common among renal cell carcinoma (RCC) patients. Herein, a novel mechanism that HOOK1 inhibits RCC growth, angiogenesis, and sunitinib resistance via TGF‐*β* and TNFSF13B/VEGF‐A axes. Furthermore, an agonist of HOOK1, meletin, is screened and showed antitumor activity through combined immunotherapy.

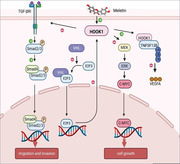

## 1 Introduction

Clear cell renal cell carcinoma (ccRCC) is the most common renal cell carcinoma subtype and one of the most aggressive histologies. So far, surgery remains the most effective clinical treatment for renal cell carcinoma, but more than 30% of ccRCC patients progress to recurrence and/or metastasis after surgery, which have a poor prognosis.^[^ ^1^ ^]^ Therefore, an in‐depth understanding of the pathogenic mechanism of ccRCC is urgently needed, and exploring targeted therapy with low toxicity and high survival rate has become the top priority in the treatment for metastatic renal cell carcinoma (mRCC).

The HOOK gene was first reported in Drosophila melanogaster nearly a century ago, but only in the past decade scientists revealed that HOOK belonged to a new and highly conserved protein family.^[^ ^2^, ^3^ ^]^ Thus far, accumulating evidence indicated that HOOK1, as well as its homologues HOOK2 and HOOK3, effected different cellular functions and participated in pathological processes. The aberrant expression of HOOK1 has been demonstrated in multiple malignancies, including ovarian cancer, breast cancer, lung cancer, and hepatocellular carcinoma.^[^ ^4^, ^5^, ^6^, ^7^ ^]^ Moreover, more than 70 missense mutations involving HOOK1 have been identified in solid tumors.^[^ ^8^ ^]^ However, the biological role and molecular mechanism of HOOK1 during RCC metastasis remains unexplored.

TNFSF13B, also known as BAFF, is a member of tumor necrosis factor superfamily, was originally reported to play a key role in lymphocyte maturation. The functional significance of elevated TNFSF13B in autoimmune disease has prompted the development of anti‐TNFSF13B monoclonal antibodies, such as belimumab, which has already been approved in the treatment of systemic lupus erythematosus.^[^ ^9^ ^]^ However, TNFSF13B has also been reported to exert a pivotal role in several other diseases, including neoplasia, such as breast cancer, glioblastoma, melanoma, and adrenocortical carcinoma.^[^ ^10^, ^11^, ^12^, ^13^ ^]^ Moreover, TNFSF13B is reported to enriched in tumor with hyperplastic blood vessels, and regarded as a biomarker for the metastases.^[^ ^14^, ^15^ ^]^ These data reinforced that TNFSF13B may play a key role in the progression of renal cell carcinoma, as RCC is strongly vascularized.

In present study, we showed that HOOK1 deletion were highly predictive for different stages of ccRCC progression and was associated with unfavorable prognosis. HOOK1 overexpression substantially suppressed RCC cell proliferation, metastasis, and angiogenesis both in vitro and in vivo. We further revealed that HOOK1 is transcriptionally regulated by nuclear factor E2F3 in VHL dependent manner. To extend these analyses, we performed a series of molecular docking screen and identified meletin may be a potential HOOK1 agonist with antitumor activity in RCC cells. Mechanistically, HOOK1 inhibited tumor growth and metastasis via canonical and non‐canonical TGF‐*β* pathway, and inhibited RCC angiogenesis and sunitinib resistance via TNFSF13B/VEGF‐A signaling. Moreover, we showed that HOOK1 could combine with anti‐PD‐1 therapeutics to enhance antitumor activity via tumor microenvironment remodeling. Further, our findings suggested that HOOK1 and its associated signaling pathway might be therapeutic targets for RCC treatment.

## 2 Results

### 2.1 HOOK1 Is Downregulated in RCC and Correlates with Better Outcomes in RCC Patients

**Figure 1:**
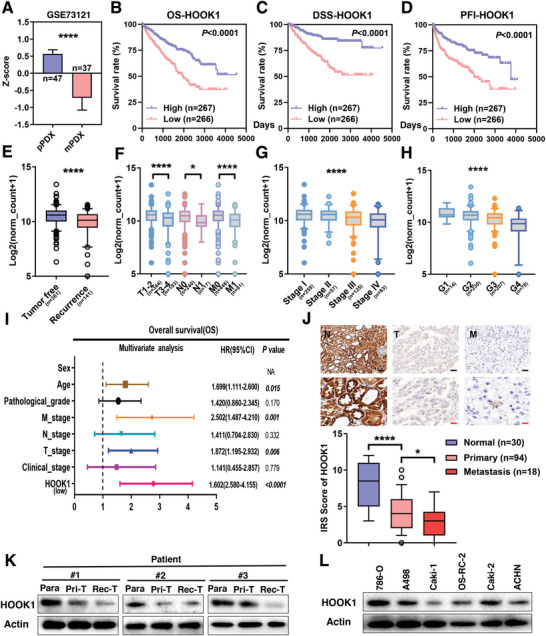
Decreasing expression of HOOK1 is associated with poor prognosis in RCC. a) Analysis of HOOK1 mRNA expression in paired PDX‐primary and PDX‐metastasis RCC tissues from Gene Expression Omnibus datasets (GSE73121). HOOK1 expression was significantly associated with b) overall survival, c) disease‐specific survival, and d) progression‐free interval in the TCGA‐KIRC cohort according to Kaplan–Meier analysis. e) The association between HOOK1 expression and tumor recurrence status, f) pathologic TNM stage, g) clinical stage, and h) grade in TCGA‐KIRC specimen. i) Forest plot showed the association between clinical parameters, HOOK1 expression and OS survival using multivariate analyses. j) Representative immunohistochemical images (upper panel) and staining index (lower panel) of HOOK1 in normal kidney tissue (*n* = 30), non‐metastatic RCC tissue (*n* = 94), and metastatic RCC tissue (*n* = 18). Scale bars: black, 200 µm; red, 50 µm. k) Immunoblotting assay of HOOK1 expression in three paired RCC adjacent normal tissues (Para), primary tumor (Pri‐T), and recurrence samples (Rec‐T). l) Protein expression of HOOK1 in primary‐derived and metastasis‐derived RCC cell lines (*p < 0.05 and ****p < 0.0001).

Accumulating studies have demonstrated that metastasis play vital roles in renal cell carcinoma progression and drug resistance. In this study, by screening genes that inhibit metastasis, we overlapped two independent public gene sets (EMTome and GSE73121),^[^ ^16^, ^17^ ^]^ among which two genes (HOOK1 and MAL2) were selected based on their significant differences in expression in metastatic patient derived xenograft (mPDX) compared with primary patient derived xenograft (pPDX) tissues (**Figure** **1**A; Figure S1A, Supporting Information). Next, Kaplan–Meier survival analysis and log‐rank tests were conducted to assess whether the overall survival (OS), disease‐specific survival (DSS), and progression‐free interval (PFI) of patients were associated with HOOK1 or MAL2 expression in TCGA‐KIRC database. Interestingly, only patients with high level of HOOK1 (not MAL2) mRNA expression had better OS (*p* < 0.0001), DSS (*p* < 0.0001), and PFI (*p* < 0.0001) than those with low mRNA expression (Figure 1B–D; Figure S1B–D, Supporting Information). What is more, pan‐cancer analysis showed HOOK1 was downregulated in most metastatic specimens (Figure S1E, Supporting Information). Taking these results together, we focused on HOOK1 for further study. Further analysis found patients with high HOOK1 mRNA expression exhibited a lower risk of recurrence (Figure 1E) and negatively correlated with tumor grade and stage (T stage, *p* < 0.0001; N stage, *p* = 0.036; non‐metastasis/metastasis, *p* < 0.0001; TNM stage, *p* < 0.0001; Clinical stage, *p* < 0.0001; Histological grade stage, *p* < 0.0001) (Figure 1F–H). Moreover, univariate and multivariate by Cox proportional hazard model was applied to determine the prognostic value of HOOK1 which showed that the high expression of HOOK1 was an independent predictor of better overall survival in patients with renal cancer (Figure 1I; Figure S1F, Supporting Information). According to the above multivariate analysis, we further constructed a nomogram model based on HOOK1 mRNA expression to predict OS 1, 3, and 5 years after RCC surgery (Figure S1G, Supporting Information). Calibration plots revealed that the nomograms were favorable to predict patient survival according to a conceptual model (Figure S1H, Supporting Information).

To valid the clinical significance of HOOK1, immunohistochemistry (IHC) analysis showed that staining score of the HOOK1 protein in metastatic RCC tissues was lower in comparison with primary RCC tissues and/or adjacent non‐tumorous tissues (Figure 1J). Additionally, three paired independent samples were tested and found that HOOK1 protein levels showed a decreasing trend compared to matched adjacent non‐tumor tissue, primary tumor, and recurrence samples, which were all consistent with TCGA analysis results (Figure 1K). Moreover, metastasis‐derived cell lines (Caki‐1, OS‐RC‐2, and ACHN) exhibited lower levels of HOOK1 than primary‐derived cell lines (786‐O, A498, and Caki‐2), respectively (Figure 1L). These results indicated that HOOK1 might play a critical role in RCC development and progression.

### 2.2 HOOK1 Inhibits RCC Proliferation, Metastasis, and Angiogenesis

**Figure 2:**
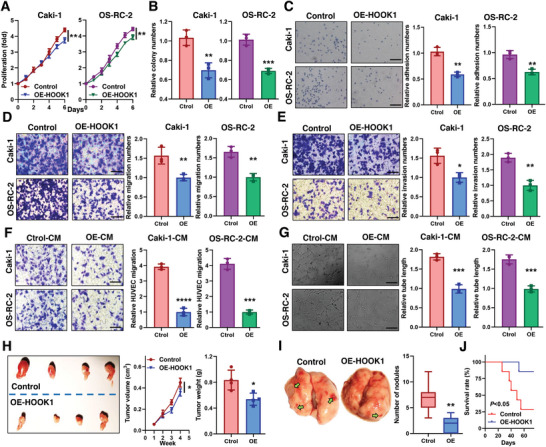
HOOK1 suppressed the proliferation, metastasis and angiogenesis of RCC cells in vitro. a) Viability of Caki‐1 and OS‐RC‐2 cells proliferation after HOOK1 overexpression was assessed by CCK‐8 assay at indicated times. b) Quantification of the colony numbers. c) The adhesive properties of the cells were analyzed with the fibronectin adhesion assay. Transwell assays were performed in transfected Caki‐1 and OS‐RC‐2 cells to evaluate d) cell migration and e) invasion ability. f) Effect of HOOK1 expression in RCC cells on migration ability of HUVECs in transwell assay. g) Representative capillary tubule structures were shown for HUVECs treated with culture medium collected from the indicated RCC cells. h) Subcutaneous xenografts (*n* = 4, each group) from the control group and HOOK1 group excised from nude mice (left panel). Tumor growth was summarized using a line chart (middle panel), while mean tumor weights were shown in the histogram (right panel). i) Representative lung nodules (green arrow; left panel) and calculated nodule numbers (right panel) from the indicated mice groups, and j) Kaplan–Meier analysis of the effect of HOOK1 on survival in the indicated mice groups (*n* = 8, each group). Data are means ± SD, *p < 0.05; **p < 0.01; ***p < 0.001, and ****p < 0.0001.

To determine the impact of HOOK1, we established stable expression of HOOK1 in Caki‐1 and OS‐RC‐2 cell lines (Figure S2A, Supporting Information). The growth curve assay revealed exogenous introduction of HOOK1 dramatically inhibited Caki‐1 and OS‐RC‐2 proliferation relative to the control group (**Figure** **2**A). These results were further confirmed by colony formation assay (Figure 2B). As adhesion of tumor cells to the extracellular matrix is a key step in cancer metastasis, we then performed cell fibronectin adhesion assays and found HOOK1 significantly suppressed cell adhesion (Figure 2C). Likewise, trans‐well assay indicated obvious suppression of migration and invasion in HOOK1‐overexpressing cells (Figure 2D,E).

Tumor‐associated angiogenesis is a rate‐limiting process in metastasis of cancer, especially in RCC. To assess whether HOOK1 effected the angiogenic behavior of vascular endothelial cells, a co‐culture system including human umbilical vein endothelial cells (HUVECs) and conditioned medium (CM) derived from Caki‐1 and OS‐RC‐2 cell lines was employed. The migration and tube formation assays revealed that overexpressing HOOK1 strongly abrogated the ability of RCC cells in migration and tube formation in HUVECs (Figure 2F,G), suggesting that HOOK1 inhibited RCC‐associated angiogenesis in vitro. In addition, xenograft models were created by subcutaneously injecting RCC cells into the flanks of nude mice. Tumor volumes and weights were smaller in the HOOK1‐overexpressing group than the control group (Figure 2H). Tail vein metastasis model revealed that induced HOOK1 decreased the number of lung metastasis compared with that in the control group (Figure 2I). Moreover, mice injected with HOOK1‐overexpressing cells had a significantly higher survival rate (survival rate analysis of HOOK1 vs control mice: 85.7% vs 28.5%; *p* = 0.027) (Figure 2J). Collectively, these data suggested that HOOK1 was a promising target for preventing metastasis in RCC.

### 2.3 VHL Regulates HOOK1 in RCC Cells in an HIF‐Independent Manner

**Figure 3:**
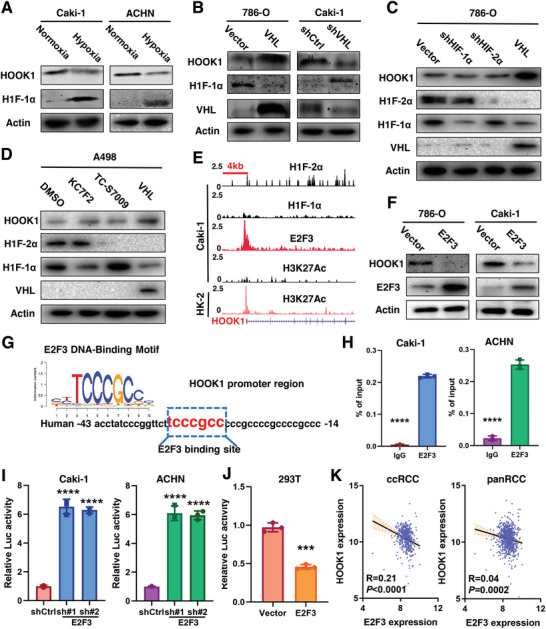
Transcriptional regulation of HOOK1 in RCC. a) The protein level of HOOK1 was measured in the indicated RCC cells under normoxia or hypoxia. b) Immunoblotting analysis of HOOK1 expression in 786‐O cell line with overexpressing VHL or in Caki‐1 cell line with decreasing VHL. c) Immunoblotting analysis of HOOK1 expression in 786‐O cell line transfected with specific shRNA against HIF‐1*α*, HIF‐2*α*, or stably expressing VHL. d) The A498 cells were pretreated with KC7F2, an HIF‐1*α* specific inhibitor; or with TC‐S 7009, an HIF‐2*α* specific inhibitor; and analyzed by western blotting with the indicated antibodies. e) ChIP‐seq analysis of HIF‐1*α*, HIF‐2*α*, E2F3, and H3K27ac enrichment at the HOOK1 promoter in Caki‐1 RCC cell line; and H3K27ac enrichment in control HK2 normal renal tubular epithelial cell line. f) Western blot analysis of HOOK1 protein level in indicated cells with E2F3 overexpression. g) E2F3 DNA‐binding sites are present in the human HOOK1 promoter region. h) Chromatin immunoprecipitation‐polymerase chain reaction (ChIP‐PCR) in Caki‐1 and ACHN cells. i) Relative HOOK1 luciferase promoter activity with E2F3 depletion in RCC cells or j) overexpression in 293T cell line. k) E2F3 was negatively associated with HOOK1 mRNA expression in TCGA‐KIRC (left panel) and pan‐RCC data (right panel).

The importance of HOOK1 in RCC prompted us to identify the regulatory mechanism responsible for its decreased expression. We first investigated HOOK1 mRNA mutations and/or copy loss in TCGA dataset, especially in ccRCC cohort, but not statistically significant when compared with other tumor types (Figure S2B,C, Supporting Information). Since a majority of RCCs were characterized by the constitutive activation of HIF1/2*α* caused by VHL inactivation, we investigated the relationship between HOOK1 and VHL‐HIF1/2*α* pathway. As shown in Figure S2D,E, Supporting Information, there was a significantly positive correlation between VHL and HOOK1 transcript abundances in ccRCC and pan‐RCC cohort. Consistently, the protein level of HOOK1 was significantly reduced under hypoxia in VHL wide‐type RCC cell lines Caki‐1 and ACHN (**Figure** **3**A). Further, we found that VHL inhibition led to significantly decreased HOOK1 mRNA and protein expression in Caki‐1 cell line, while ectopic VHL augmented the expression of HOOK1 in VHL‐mutant RCC 786‐O and A498 cell line (Figure 3B,D; Figure S2F,G, Supporting Information). Taken together, downregulated HOOK1 expression was associated with VHL inhibition or mutation in RCC.

To delineate the molecular mechanism of VHL‐dependent HOOK1 change, we speculated that high level of HIF‐1*α* and/or HIF‐2*α* might downregulate the expression of HOOK1. However, downregulation HIF‐1*α* or HIF‐2*α* with either lentivirus or specific inhibitor (KC7F2: HIF‐1*α* inhibitor; TC‐S7009: HIF‐2*α* inhibitor) could not change HOOK1 expression level (Figure 3C,D). More importantly, chromatin immunoprecipitation followed by deep sequencing (ChIP‐seq) in Caki‐1 cells did not identify notable HIF‐1*α* or HIF‐2*α* binding peaks in the gene loci of HOOK1, indicating that HOOK1 was not the direct transcriptional targets of HIF‐1*α* or HIF‐2*α* (Figure 3E). In other words, VHL might regulate the transcription of HOOK1 in RCC cells in HIF‐independent manner.

### 2.4 E2F3 Mediates Regulation of HOOK1 by VHL

As the above analysis excluded the possibility of direct regulation of HOOK1 by HIF‐1*α* or HIF‐2*α*, we then turned our attention to E2F3, which was previously reported been regulated by VHL.^[^ ^18^, ^19^ ^]^ Western blotting confirmed that E2F3 were significantly downregulated in RCC cells with ectopic VHL transfection (Figure S3A, Supporting Information). Besides, Kaplan–Meier survival analysis revealed that E2F3 was positively associated with worse prognosis in RCC patients (Figure S3B, Supporting Information). As expected, overexpression of E2F3 could inhibit HOOK1 mRNA and protein expression (Figure 3F; Figure S3C, Supporting Information). E2F3 enrichment peaks was identified at the promoter region of HOOK1 with diminished histone acetylation at this same region with the help of ChIP‐seq (Figure 3E). Additionally, bioinformatics analysis revealed a highly conserved, putative E2F3 binding sequence, TCCCGCC, located −38 bp to −32 bp upstream of human HOOK1 transcription start site (Figure 3G). Furthermore, Chromatin immunoprecipitation‐polymerase chain reaction ChIP‐PCR assay confirmed the binding of E2F3 to the HOOK1 promoter in RCC cells (Figure 3H). Luciferase promoter assay showed that E2F3 suppression increased HOOK1 activity in Caki‐1 and ACHN cell lines (Figure 3I), while E2F3 overexpression decreased HOOK1 transcription in HEK293T cell (Figure 3J). This negative correlation was also validated in TCGA ccRCC and pan‐RCC database (*r* = 0.21, *p* < 0.0001; *r* = 0.04, *p* = 0.0002, respectively) (Figure 3K). Collectively, these results suggested that E2F3 regulated HOOK1 transcription though directly binding to the HOOK1 promoter via VHL in RCC.

### 2.5 Meletin Is a Potential HOOK1 Agonist with Antitumor Activity in RCC Cells

**Figure 4:**
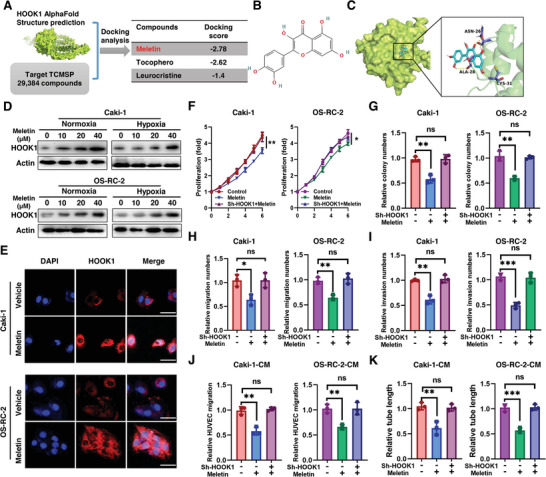
Antitumor activity of HOOK1 agonist meletin in RCC. a) Schematic overview of the virtual screening approach based on Chinese medicine monomer library (TCMSP). b) Chemical structure of meletin. c) Predicted model of meletin binding to the CH domain of HOOK1 as shown by computational docking. d) Protein expressions of HOOK1 in both meletin‐treated Caki‐1 and OS‐RC‐2 cell lines under either normoxic or hypoxic condition. e) Representative confocal images of in vitro RCC cells with or without meletin. f) Effect of meletin on the cell viability of HOOK1 knockdown in Caki‐1 and OS‐RC‐2 cell lines. g) Effect of meletin on the colony forming ability. Effect of h) meletin on the migration and i) invasion of HOOK1 knockdown in RCC cell lines. Determination of j) RCC‐derived migration and k) tube formation of HUVECs via either HOOK1 knockdown or co‐treatment meletin.

Since E2F3 was considered “undruggable” due to its significant structural disorder and lack of defined small‐molecule binding pockets, we conducted a structure‐based virtual screen of ≈2.9 thousand compounds using TCMSP database in order to identify HOOK1 agonist.^[^ ^20^, ^21^ ^]^ Based upon binding mode analysis, meletin was selected, which targets with absolute highest docking score (**Figure** **4**A,B). By examining active sites, we found meletin formed hydrogen bonds with Asn26/Ala28/Cys31 at the CH domain of HOOK1 (Figure 4C). Moreover, a dose‐dependent accumulation of the HOOK1 protein was detected in RCC cells after treatment with different concentration of meletin (0, 10, 20, and 40 µm) for 48 h under either normoxic or hypoxic condition (Figure 4D). Also, the immunofluorescence signaling intensities of HOOK1 in both Caki‐1 and OS‐RC‐2 cell lines had an upward trend after meletin treatment (Figure 4E). To further analyze how meletin regulated HOOK1 activity, rescue experiments were performed by treating RCC cells with meletin with/without HOOK1 knockdown. We found that meletin treatment could impair the effect of sh‐HOOK1 on promoting growth and metastasis of RCC cells (Figure 4F,I; Figure S3D, Supporting Information). Furthermore, the anti‐RCC angiogenesis effect of meletin was validated by HUVECs tube formation and migration assay in co‐culture system. Migrated HUVECs and skeletonized tube‐like structures were found decreased in meletin group, but this biological effect was abrogated by HOOK1 knockdown (Figure 4J,K). Taken together, these results uncovered that meletin might be a potential HOOK1 agonist with antitumor activity in RCC cells.

### 2.6 HOOK1 Inhibits Tumor Growth and Metastasis via Canonical and Non‐Canonical TGF‐*β* Pathway

**Figure 5:**
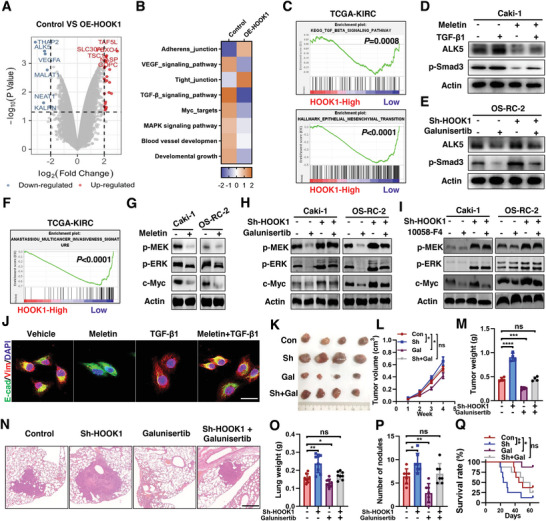
HOOK1 inhibits the TGF‐*β* canonical and noncanonical pathway. a) Volcano plot of the differentially expressed mRNAs between untreated cancer cells and overexpression of HOOK1. Red and blue dots indicate the up‐ and downregulation of mRNAs, respectively. b) RNA‐seq of HOOK1 overexpressing cells were analyzed by KEGG and GO biological process. c) GSEA plots demonstrating the enrichment of gene sets in the ranked gene list of HOOK1 down versus HOOK1 up available from TCGA‐KIRC specimen cohorts. d) Protein levels of ALK5 and p‐Smad3 in Caki‐1 cell under co‐treatment of meletin and TGF‐*β*1. e) Protein levels of ALK5 and p‐Smad3 in OS‐RC‐2 cell under treatment of galunisertib and/or with HOOK1 knockdown. f) GSEA enrichment plots. g) Protein expression level changes of p‐MEK, p‐ERK, and c‐Myc in the Caki‐1 and OS‐RC‐2 cells treated with meletin. h) Immunoblotting analysis of MAPK pathway molecules in HOOK1‐knockdown and control cells with or without galunisertib treatment. i) Immunoblotting analysis of MAPK pathway molecules in HOOK1‐knockdown and control cells with or without 10058‐F4 treatment. j) Immunofluorescence analysis of EMT marks (E‐cadherin, green; Vimentin, red) in treated cells; Scale bar, 20 um. Representative figures of subcutaneous xenografts from mice with k) various treatments; l) line chart showing tumor growth and m) histogram showing tumor weight. n) Representative H&E‐stained lung sections; histogram showing o) lung metastatic nodules and p) lung weight. q) Kaplan–Meier analysis of the effect of galunisertib and/or HOOK1‐knockdown on cumulative survival in the indicated mice groups (*n* = 8, each group). *p*‐value was determined by log‐rank test.

To further explore the specific mechanism underlying the effect of HOOK1 on renal cancer, RNA‐seq was performed in Caki‐1 cell line. The differences in gene expression between control group and HOOK1 stable overexpression group were visualized by volcano plots (**Figure** **5**A). Among them, several key tumor‐induced genes, such as TGF‐*β*RI/ALK5 and VEGF‐A, demonstrated the precision of our method for screening DEGs. In fact, growth factor‐*β* (TGF‐*β*) signaling and epithelial‐mesenchymal transition (EMT) pathway were enriched by GO functional and KEGG analysis (Figure 5B), which were further validated by gene set enrichment analysis (GSEA) in TCGA cohort (Figure 5C). Considering the important and complicated role of TGF‐*β* signaling in tumorigenesis and metastasis, the relationship between HOOK1 and TGF‐*β* signaling attracted our strong interest to uncover. As expected, meletin treatment could hinder ALK5 and p‐Smad3 expression induced by TGF‐*β*1 (Figure 5D). On the contrary, galunisertib, a clinically relevant small molecule inhibitor of ALK5, could block the stimulatory effects of ALK5/p‐Smad3 expression activated by HOOK1 depletion (Figure 5E). All of the results indicated that HOOK1 inhibited the canonical TGF‐*β*/ALK5/p‐Smad3 pathway.

In addition, multi‐cancer invasive pathway and MAPK signal as well as Myc targets were downregulated in HOOK1 higher group, respectively in GSEA analysis in TCGA patient cohort and cell RNA‐seq analysis (Figure 5B,F). Indeed, luciferase analysis indicated that the transactivity of Myc significantly reduced in HOOK1 ectopic expression group (Figure S4A, Supporting Information). Besides, the mRNA level of several Myc target genes including MALAT1 and NEAT1 were decreased following HOOK1 induction (Figure 5A). Furthermore, immunoblotting showed meletin induced HOOK1 expression, leading to inhibition of the activation of phospho‐extracellular signal‐regulated kinase (p‐ERK), phospho‐MEK (p‐MEK), and c‐Myc (Figure 5G). As TGF‐*β* was a well‐known regulator for tumor development, we investigated whether MAPK and Myc were potential downstream of HOOK1 via atypical TGF‐*β* signaling. Importantly, treatment of ALK5 inhibitor profoundly reduced the increased p‐ERK, p‐MEK, and c‐Myc expression caused by HOOK1 knockdown in RCC cells (Figure 5H). On the contrary, c‐Myc inhibitor, 10058‐F4, did not cause this effect (Figure 5I). These observations suggested that HOOK1 could also blunt the noncanonical TGF‐*β*/MEK/ERK/c‐Myc signal.

To investigate whether altered TGF‐*β* pathway played a role in the inhibition of RCC proliferation and metastasis by HOOK1, we stimulated RCC cells overexpressing HOOK1 with the ALK5 agonist TGF‐*β*1. As expected, ectopic HOOK1 expression obviously abolished the promoting effects of TGF‐*β*1 on RCC cell growth (Figure S4B,C, Supporting Information), migration and invasion (Figure S4D–F, Supporting Information). Consistently, immunofluorescence confirmed that HOOK1 significantly inhibited the expression of EMT markers in CAKI‐1 cells induced by TGF‐*β*1 (Figure 5J). Consistent with the in vitro effects, the enhanced tumor cell growth and weight after HOOK1 knockdown could be rescued in vivo by the addition of galunisertib (Figure 5K–M). In addition, galunisertib treatment notably reduced the lung metastasis nodes and weight resulted from depletion of HOOK1, thus prolonging survival in tumor‐bearing nude mice (Figure 5N–Q). Altogether, these data indicated that HOOK1 inhibited tumor growth and metastasis via canonical and non‐canonical TGF‐*β* pathway.

### 2.7 HOOK1 Inhibits RCC Angiogenesis and Sunitinib Resistance via TNFSF13B/VEGF‐A Signaling

**Figure 6:**
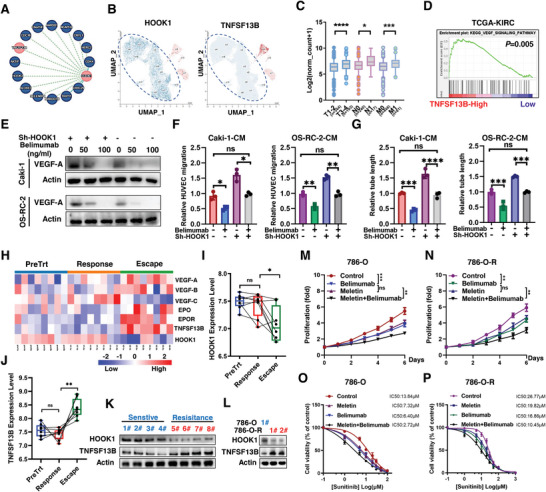
HOOK1 attenuates tumor angiogenesis and sunitinib resistance and by targeting TNFSF13B. a) Circle‐plot showing mass spectrometry results of IPs from HEK293 cells expressing Flag‐HOOK1. b) UMAP analysis showing scRNAseq data of different cells in human kidney form proteinatlas database. Renal proximal tubule epithelial cells express a high level of HOOK1 but do not express TNFSF13B. c) The association between TNFSF13B expression and pathologic TNM stage. d) GSEA enrichment plots. e) The protein level of VEGF‐A in RCC cells with HOOK1 knockdown and/or treated with belimumab at different concentrations. f,g) Assessment of the RCC‐derived migration and tube formation of HUVECs via HOOK1 knockdown and/or treated with belimumab. h) Heatmap analysis of angiogenesis‐related genes in pre‐treatment phase, sunitinib response, and escape in GSE76068. i) HOOK1 and j) TNFSF13B expression in pre‐treatment, sunitinib response, and escape phase. k) The protein level of HOOK1 and TNFSF13B from RCC patients with (*n* = 4) or without (*n* = 4) sunitinib resistance. l) The protein level of HOOK1 and TNFSF13B in 786‐O and 786‐O‐R (sunitinib resistance) cells. Viability of m) 786‐O and n) 786‐O‐R cells treated as indicated. Dose‐response curves for sunitinib in o) 786‐O and p) 786‐O‐R cells treated as indicated.

Since RNA‐seq showed that the key angiogenic factor, VEGF‐A, was downregulated in HOOK1 overexpression cells compared with control cells (Figure 5A), we then explored whether HOOK1 mediated RCC angiogenesis by inhibiting VEGF‐A via TGF‐*β* signal. However, immunoblotting showed ectopic ALK5 could not abolish the inhibition of VEGF‐A by meletin (Figure S5A, Supporting Information). Similarly, although the RCC culture medium overexpressing ALK5 promoted HUVECs’ migration and/or tube formation to some extent, ALK5 did not eliminate the angiogenic inhibitory effect of HOOK1 (Figure S5B,C, Supporting Information), suggesting that HOOK1 might inhibit RCC angiogenesis through other mechanisms. Then, mass spectrometry (MS) assay was performed to identify candidate protein(s) that co‐precipitated with HOOK1. TNFSF13B, which enriched in tumor with hyperplastic blood vessels,^[^ ^14^ ^]^ was identified as a possible HOOK1‐associated protein (**Figure** **6**A). Intriguingly, single‐cell sequencing dataset from the proteinatlas (https://www.proteinatlas.org/) revealed that TNFSF13B was barely expressed in human renal proximal tubule epithelial cells, from which most RCCs originated,^[^ ^22^, ^23^ ^]^ when compared with HOOK1 (Figure 6B; Figure S5D, Supporting Information). But TNFSF13B enrichment in tumor samples, and patients with high TNFSF13B expression were correlated with tumor progression and poor prognosis (Figure 6C; Figures S5E–J, S6A, Supporting Information). Moreover, GSEA analysis revealed that VEGF signaling pathway was significantly enriched in TNFSF13B‐higher tumors (Figure 6D), and pan‐cancer analysis manifested that expression of HOOK1 was widely elevated in many cancers and metastatic tumors (Figure S6A, Supporting Information). To evaluate this possibility, we treated HOOK1‐decreasing cells with different concentrations of belimumab, an FDA approved antibody against TNFSF13B,^[^ ^9^ ^]^ to determine whether HOOK1 diminished RCC angiogenesis progression via TNFSF13B. Western blot assays showed that VEGF‐A level decreased with the increase of belimumab concentration (Figure 6E). Furthermore, HUVECs’ migration and tube formation assays confirmed that VEGF secretion and angiogenesis induced by HOOK1 silencing could be inhibited by belimumab (Figure 6F,G). These results suggested that HOOK1‐induced inhibition of RCC angiogenesis was dependent on TNFSF13B.

Accumulating evidence demonstrates that resistance to sunitinib, a first line drug that inhibits tumor angiogenesis, has become a major problem in prolonging survival in advanced RCC.^[^ ^24^ ^]^ Interestingly, elevated VEGF‐A was one of the hallmarks of sunitinib resistance as shown by paired xenograft (PDX) sequencing in patients with sunitinib‐resistant RCC (GSE76068) (Figure 6H). Considering HOOK1 inhibited the expression of VEGF‐A through TNFSF13B, we examined whether HOOK1 and TNFSF13B were associated with sunitinib sensitivity. Intriguingly, tight clustering analysis showed that HOOK1 and TNFSF13B were not affected by sunitinib during response phase compared with pre‐treatment phase, indicating HOOK1 and TNFSF13B were not downstream genes of sunitinib. On the contrary, the activation level of HOOK1 was significantly inhibited, while TNFSF13B was dramatically upregulated during the escape phase, which were validated in patient tumors with sunitinib resistance and sunitinib‐resistant 786‐O (786‐O‐R) cells, suggesting that HOOK1 and TNFSF13B might act as key genes to reverse sunitinib resistance (Figure 6H–L). To test this hypothesis, the sunitinib sensitivity of 786‐O and sunitinib resistance of 786‐O‐R cell lines were evaluated with meletin and/or belimumab treatment. It was clear that using meletin or belimumab could increase 786‐O cell sunitinib sensitivity and decrease sunitinib resistance of 786‐O‐R, and the synergetic effect of meletin and belimumab was more significant as compared to the single agent (Figure 6M,N). Similarly, the median IC50 values were notably lower than those in the control after meletin and/or belimumab treatment (Figure 6O,P). Together, we found that genetic and pharmaceutical of HOOK1/TNFSF13B/VEGF‐A axis inhibited RCC angiogenesis and sensitized sunitinib.

### 2.8 HOOK1 Binds to TNFSF13B and Induces Its Degradation in RCC Cells

**Figure 7:**
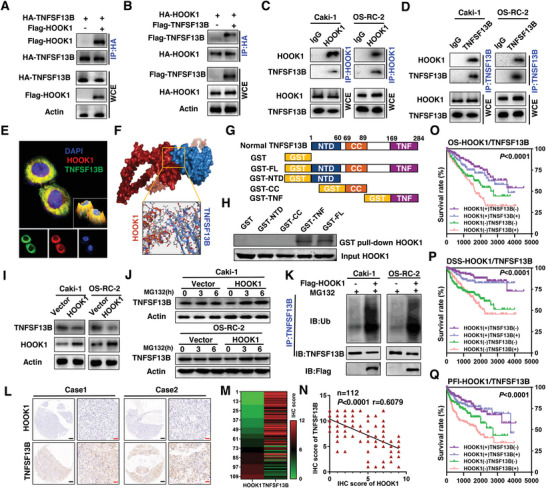
HOOK1 promotes the degradation of TNFSF13B. Immunoprecipitation were performed using anti‐HA agarose on lysates derived from 293T cells exogenously expressing a) Flag‐tagged HOOK1 and HA‐tagged TNFSF13B, or b) Flag‐tagged TNFSF13B and HA‐tagged HOOK1. Co‐IP was performed to examine the relationship between c) endogenous HOOK1 and d) TNFSF13B in RCC cells. e) Caki‐1 cells were immunostained for TNFSF13B (in green) and HOOK1 (in red); yellow in the merged magnified images (upper) indicates the co‐localization. A 3D visualization was shown in the bottom right. f) Graphical representation of 3D structures of the docking models of HOOK1 with TNFSF13B, and zoom‐in images showing the interaction interface of amino acid in the binding site. g) A schematic diagram of GST fusion constructs. GST‐FL, full‐length TNFSF13B; NTD, N‐terminal domain; CC, coiled coil; TNF, TNF_2 domain. h) Binding of TNFSF13B domains with HOOK1. Input: reaction aliquots collected before the pull‐down reaction and analyzed in parallel with the samples using Western blotting with an anti‐human HOOK1 antibody. i) Western blotting was performed to examine the expression of TNFSF13B in RCC cells expressing HOOK1. j) The expression level of TNFSF13B was effectively rescued after proteasome inhibitor MG132 treatment. k) Detection of TNFSF13B ubiquitination by immunoprecipitated and immunoblotted as indicated. l) Representative images of TNFSF13B expression from the same sample slices used for examining HOOK1 expression by immunohistochemistry analysis. m) The expression levels of HOOK1 and TNFSF13B are shown in the heatmap. n) Scatterplot of expression scores of HOOK1 versus TNFSF13B with a regression line showing a negative correlation. Prognostic value of combining HOOK1 and TNFSF13B levels was analyzed by Kaplan–Meier analysis in TCGA‐KIRC samples; o) overall survival, p) disease‐specific survival, and q) progression‐free interval.

Co‐immunoprecipitation (co‐IP) experiments were conducted to verify the interaction between HOOK1 and TNFSF13B as the mass spectrometry (MS) indicated (Figure 6A). Exogenously expressing Flag‐tagged HOOK1 or TNFSF13B could be eluted with HA‐tagged TNFSF13B or HOOK1, respectively (**Figure** **7**A,B). Moreover, TNFSF13B was efficiently precipitated with endogenous HOOK1 and vice versa when examined in Caki‐1 and OS‐RC‐2 cell lines (Figure 7C,D). Further immunofluorescence‐based co‐expression assays showed the green fluorescence representing TNFSF13B was superimposed with the red fluorescence representing HOOK1, which also suggested a strong co‐localization between HOOK1 and TNFSF13B (Figure 7E). To determine the interfacial interaction of sequences between HOOK1 and TNFSF13B, computational structural modeling was analyzed, and demonstrated through a protein–protein molecular docking experiment (Figure 7F). To verify the specificity of this interaction, a series of deletion was constructed based on the secondary structure of TNFSF13B with GST pull‐down indicating that HOOK1 bound to the TNF domain of TNFSF13B (Figure 7G,H).

As TNFSF13B could bind to HOOK1, we then detected the effect of HOOK1 on TNFSF13B expression. However, TNFSF13B protein level, but not mRNA level, was markedly decreased in HOOK1‐overexpressing cells compared with control cells (Figure 7I; Figure S6B, Supporting Information). Further, in Caki‐1 and OS‐RC‐2 cell lines in the presence of autophagy lysosome inhibitor ammonium chloride(NH4Cl) or ubiquitin‐proteasome inhibitor MG132, results showed that TNFSF13B protein was not changed significantly in HOOK1‐overexpressing cells treated with NH4C1, but was effectively rescued after MG132 treatment when compared to the control, suggesting that HOOK1 promoted the degradation of TNFSF13B through the proteasome pathway, rather than the lysosome‐dependent pathway (Figure 7J; Figure S6C, Supporting Information). Further cycloheximide chase analysis also revealed that TNFSG13B was degraded faster when co‐expressed with HOOK1 (Figure S6D, Supporting Information). What is more, reintroduction of HOOK1 in RCC cells led to a profound decrease in TNFSF13B protein, together with an increase in TNFSF13B polyubiquitination (Figure 7K). These observations indicated that HOOK1 affected TNFSF13B expression through ubiquitination mechanisms. Besides, an inverse correlation between HOOK1 and TNFSF13B protein was also observed in tissue microarrays (TMAs) consisting of 122 RCC samples (*r* = −0.4XX, *p* < 0.0001; Figure 7L–N). More importantly, Kaplan–Meier survival analysis revealed that HOOK1‐high/TNFSF13B‐low subgroup had best OS, DSS and PFI, while the HOOK1‐low/TNFSF13B‐high subgroup suffered the worst outcome (Figure 7O–Q). Together, our results demonstrated that the HOOK1 governed TNFSF13B stability and played a critical role in disease progression in renal cell carcinoma.

### 2.9 Targeting HOOK1‐TNFSF13B Axis Substantially Improves Sunitinib Efficacy In Vivo

**Figure 8:**
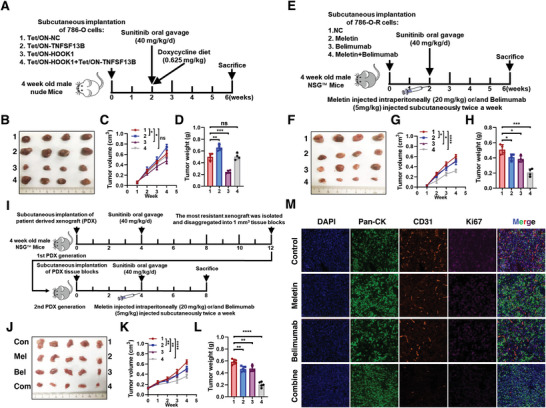
HOOK1/TNFSF13B axis inhibited tumor angiogenesis and sunitinib resistance in vivo. a) Application of Tet‐On systems for doxycycline‐inducible HOOK1 and TNFSF13B expression. On week 2, the tumor‐bearing nude mice were treated with sunitinib and doxycycline diet for 4 weeks. b) Photograph and comparison of excised tumor size. c) Tumor volumes recorded on the indicated days are shown. d) The tumor weights of the indicated group were measured. e) Schematic illustration of experimental design of 786‐O‐R cells subcutaneously injected in NSG mice were treated with meletin and/or belimumab to abrogate sunitinib resistance. Photograph of the f) excised tumors, and g) statistical analysis of the tumor volumes and h) weights. i) Illustration of the methodology used to establish RCC sunitinib resistance PDX models and treated with meletin and/or belimumab to abrogate sunitinib resistance. Representative j) tumor photo, k) tumor growth curve, and l) statistical results of tumor weight. m) PDX tumor sections derived from the indicated groups were stained. Cancer cells (pan‐cytokeratin, green), blood vessels (CD31, red), proliferation marker (Ki‐67, purple), and nucleus (DAPI, blue).

To support the above in vitro findings, we used three different mouse models to test the potential clinical application of the HOOK1‐TNFSF13B axis. In the first model, we employed a Tet‐on inducible system in 786‐O cells injected into nude mice, addition of doxycycline (Dox) induced HOOK1 and/or TNFSF13B expression, and treatment with sunitinib as schematic diagram shown (**Figure** **8**A). It was found that HOOK1 overexpression could reduce the TNFSF13B transduced tumor burden and increased the sensitivity of RCC to sunitinib (Figure 8B–D). In the second mouse model, NSG mice were subcutaneously injected with 786‐O‐R cells (Figure 8E). Similarly, restoration of HOOK1 by meletin or blockade of TNFSF13B through belimumab could inhibit sunitinib resistance. At the same time, synergetic effects of meletin and belimumab were significant as compared to the single agent (Figure 8F–H). Third, we established a sunitinib‐resistant PDX model (Figure 8I), and found that the combination treatment had better antitumor activity than meletin or belimumab alone, which was evident by the reduced PDX‐based tumor growth and tumor weight (Figure 8J–L). Immunohistochemical staining showed tumor displayed lower cell proliferation and lower angiogenesis indices after treatment (Figure 8M). Additionally, no statistically weight loss or other signs of acute or delayed toxicity were observed in any of the mice during treatment (Figure S7A,B, Supporting Information). Overall, these findings indicated that meletin and belimumab had therapeutic effects on sunitinib resistance in renal cell carcinoma, and warrants further clinical investigation for RCC therapy.

### 2.10 HOOK1 Combined with Anti‐PD‐1 Enhances Antitumor Activity via TME Remodeling

**Figure 9:**
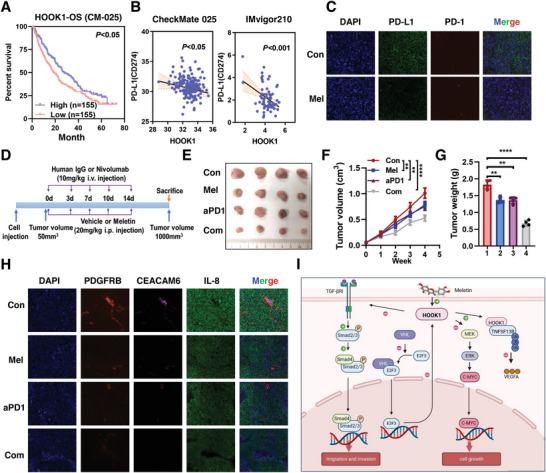
HOOK1 enhanced anti‐PD‐1 anti‐tumor activity via TME remodeling. a) Low HOOK1 expression is associated with worse overall survival in CheckMate‐025 RCC phase III trial. b) PD‐L1 expression negatively correlated with HOOK1 expression in CheckMate‐025 (left) and IMvigor210 (right) cohorts. c) Representative IF staining of PD‐L1 (red) and PD‐1(green) in Renca tumors with or without meletin treatment. d) Schematic diagram shown mice with subcutaneous Renca tumors (*n* = 4/group) as indicated. Representative e) tumor photo, f) tumor growth curve, and g) statistical results of tumor weight. h) Tumor sections derived from the indicated groups were stained. Cancer‐associated fibroblasts (CAFs) (PDGFRB, red), pro‐inflammatory factor (IL‐8, green), extracellular matrix marker (CEACAM6, purple), and nucleus (DAPI, blue). i) Model illustrating tumor suppression mechanism regulated by HOOK1 in RCC growth and metastasis.

Finally, as previous study indicated TGF‑*β* affected escape from immune surveillance^[^ ^25^ ^]^ and our above results showed HOOK1 inhibited TGF‐*β* pathway. We analyzed CheckMate 025(CM‐025) (phase 3 trial of anti‐PD‐1 in 180 advanced RCC)^[^ ^26^ ^]^ and IMvigor210(IV‐210) (Phase 2 study investigating anti‐PD‐L1 in metastatic urothelial cancer, including 66 carcinoma of renal pelvis)^[^ ^27^ ^]^ data to test whether HOOK1 enhanced anti‐tumor immunity. Patients with high HOOK1 expression indicated a significantly longer OS (**Figure** **9**A; Figure S8A, Supporting Information), better MSKCC Prognostic Score (Figure S8B, Supporting Information) in the CM‐025 cohort. Interesting, levels of HOOK1 showed a negatively correlation with tumor PD‐L1, not PD‐1 expression in both cohorts (Figure 9B; Figure S8C, Supporting Information), which were further demonstrated in meletin treated PDX tumors and murine RCC cell Renca induced tumors (Figure 9C; Figure S8D, Supporting Information). However, no association was observed between HOOK1 expression and CD8 cells infiltration in margin and center of tumor in CM‐025 cohort (Figure S8E, Supporting Information), similarly results were also found in IV‐210 (Figure S8F, Supporting Information), suggesting HOOK1 might exert immunotherapy effect through other ways. Considering tumor microenvironment (TME) was a complex ecosystem and associated with response to checkpoint inhibitor therapy, we then evaluated whether HOOK1 could reprogram TME. As expected, HOOK1 high expression group had a lower proportion of cancer‐associated fibroblasts (CAF), extracellular matrix (ECM) and pro‐inflammatory factors, and was associated with a lower EMT score (Figure S8G–J, Supporting Information), indicating HOOK1 enhanced antitumor activity via TME remodeling.

To confirm this observation, Renca cells were injected into the flank of C57BL/6 mice, followed by treated with meletin and/or nivolumab (anti‐PD‐1) (Figure 9D). As shown in Figure 9E–G, meletin, nivolumab and meletin plus nivolumab treated mice showed a delay in tumor growth, as well as the combination meletin/nivolumab group. Moreover, meletin/nivolumab group induced a significant upregulation of epithelial E‐ cadherin and lower expression of vimentin, PDGFRB (CAFs marker),^[^ ^28^ ^]^ IL‐8 (pro‐inflammatory factor),^[^ ^27^ ^]^ CEACAM6 (ECM gene)^[^ ^29^ ^]^ than single agent alone (Figure 9H; Figure S8K, Supporting Information). Together, these data corroborated our hypothesis that HOOK1 could drive tumor cells into a less mesenchymal phenotype and enhanced anti‐PD‐1 antitumor activity via TME remodeling.

## 3 Discussion

Metastasis is a complex, multistep process that requires cancer cells to acquire new phenotypes, accompanied by invasion and induction of angiogenesis.^[^ ^30^ ^]^ In recent years, scientists discovered that one way cancer cells acquire the ability to metastasize is by losing metastasis suppressor genes. In this work, we discovered that HOOK1 expression was downregulated and associated with poor prognosis in RCC. Ectopic expression of HOOK1 dramatically suppressed RCC growth, metastasis and angiogenesis both in vitro and in vivo. These results indicate that HOOK1 may be as a tumor suppressor and plays an important role in the progression and metastasis of RCC.

Von Hippel‐Lindau (VHL) tumor suppressor gene mutation has been identified as a characteristic of ccRCC.^[^ ^31^ ^]^ Our study found HOOK1 was a target gene of VHL. Considering numerous studies indicated that HIF‐a was the major substrate of VHL, we then investigated whether HIFs regulated HOOK1. Interesting, ChIP‐seq and functional studies showed HOOK1 was not a downstream target of HIFs. Meanwhile, we found E2F3 (transcription factor 3), which correlated with poor prognosis in a variety of cancers,^[^ ^32^, ^33^ ^]^ was negatively regulated HOOK1 transcription though directly binding to the HOOK1 promoter by VHL dependent manner in RCC. This finding provided a novel regulatory mechanism for HOOK1 and a new therapeutic target for HIF‐independent RCC.

Recent advances focus on understanding the molecular details of the TGF‐*β* signaling cascade, and its interactions with other signaling pathways. The TGF‐*β*/Smad canonical pathway plays a critical role in metastasis by regulating EMT, resulting in loss of cell polarity and extracellular matrix degradation. In fact, decreased cell adhesion is the molecular basis for tumor cell infiltration and metastasis. Besides, the MEK/ERK (MAPK) signaling is a fundamental pathway in cellular carcinogenesis, and c‐Myc is a transcription factor that induces multiple oncogenes and cell cycle regulators to promote the survival and proliferation of tumor cells, which means the MAPK and c‐Myc pathway are also crucial in regulating RCC cell malignant phenotype. By analyzing the RNA‐seq data and through multiple fundamental experiments, we confirmed HOOK1 inhibited the TGF‐*β*/ALK5/p‐Smad3 canonical signaling pathway and the noncanonical TGF‐*β*/MEK/ERK/c‐Myc signal, which contributed to suppress the tumor growth and metastasis in ccRCC.

As one of the hallmarks of malignancies, aberrant active angiogenesis is the main reason for therapeutic failure and poor survival in RCC. However, the mechanism underlying RCC angiogenesis are far from understood. Through proteomic study and subsequent analysis, we identified TNFSF13B, a novel target of HOOK1, was an important angiogenic signal in RCC progression and sunitinib resistance. Previous studies mainly focused on the abnormal expression of TNFSF13B that promoted the occurrence of multiple sclerosis (MS) and Systemic Lupus Erythematosus (SLE).^[^ ^34^, ^35^ ^]^ Interestingly, people with MS or SLE are at greater risk of developing cancer than the general population.^[^ ^36^, ^37^, ^38^ ^]^ More importantly, TNFSF13B is reported to be enriched in tumor with hyperplastic blood vessels.^[^ ^14^ ^]^ Consistent with these studies, our data demonstrated that high TNFSF13B expression was correlated with the tumor progression, poor prognosis, and sunitinib resistance. Mechanically, HOOK1 directly bound to the TNF domain of TNFSF13B and degraded TNFSF13B through the ubiquitin‐proteasome degradation pathway. These results provide a more in‐depth understanding of the anti‐angiogenic molecular mechanism of HOOK1/TNFSF13B/VEGF‐A axis and provide clues for antagonizing RCC sunitinib resistance.

Meletin, also known as quercetin, is a flavonoid found in abundance in many fruits and vegetables that has been shown to inhibit growth and angiogenesis in a variety of tumors. Previous studies also showed meletin could act as a sensitizer and protect non‐cancer cells from the side effects of currently used cancer therapies, and has potential synergistic effects when combined with chemotherapeutic agents or radiotherapy.^[^ ^39^, ^40^, ^41^ ^]^ However, the detailed anticancer mechanisms of meletin was still unclear. Here, through virtual screening and medicinal testing, we uncovered that meletin might act as a potential HOOK1 agonist with antitumor activity in RCC cells. More importantly, we showed that meletin could significantly decrease sunitinib resistance when combined with belimumab. Thus, combined with our present findings, both meletin and belimumab are potential drugs for chemotherapy resistance in RCC.

Immunotherapy emerges as a novel therapeutic strategy for advanced RCC patients. Disappointingly, PD‐L1 expression and CD8^+^ T cell infiltration did not reflect well the response of RCC patients to ICI.^[^ ^42^, ^43^ ^]^ Considering the tumor microenvironment (TME) may be one of the most important factors affecting the efficacy of PD‐L1/PD‐1 blockade therapy.^[^ ^44^ ^]^ We analyzed two public clinical trial studies and found meletin could enhance anti‐PD‐1 antitumor activity via TME remodeling, which was consistent with previous reports that meletin had immunologically synergistic in solid tumor.^[^ ^45^, ^46^ ^]^ Thus, although we present only limited bioinformatical data and mice studies, this result at least suggested that meletin treatment might represent a promising approach to RCC immunotherapy. However, the specific mechanism of meletin in improving TME, as well as the safety and efficacy in clinical application, requires to be further investigated.

In conclusion, our study reveals the importance of HOOK1 in retarding RCC growth and metastasis via canonical and non‐canonical TGF‐*β* pathway, and inhibiting RCC angiogenesis and sunitinib resistance via TNFSF13B/VEGF‐A signaling. Moreover, the pharmacological of meletin that reverse sunitinib resistance and promise immune effects could be considered as a novel approach for alleviating RCC condition (Figure 9J). This potential therapeutic strategy needs to be evaluated in the next future.

## 4 Experimental Section

### Chromatin Immunoprecipitation (ChIP)‐Sequencing and Analyses

ChIP‐seq was performed using a Chromatin Immunoprecipitation Assay Kit (Millipore) according to the manufacturer's protocol. All raw reads were aligned to reference genome version GCCh37/hg19 with the use of bowtie2 tool. Motif enrichment was performed using Hypergeometric Optimization of Motif EnRichment suite. ChIP assay was performed as previously described.^[^ ^47^ ^]^ Briefly, Caki‐1 and HK2 cells were washed with PBS and crosslinked with 1% formaldehyde for 10 min and then sonicated to generate 100‐ to 500‐bp DNA fragments. Soluble chromatin was precipitated with anti‐E2F3, and IgG. Specific primer sets were designed to amplify a target sequence within the human HOOK1's promoter.

### Mass Spectrometry and Protein–Protein Interaction Assays

To identify HOOK1‐bound proteins, human embryonic kidney 293T cells expressing or not expressing Flag‐HOOK1.Cell lysates were collected to perform Flag‐IP and the band was excised and then subjected to LC‐MS/MS analysis. For identification of binding proteins of HOOK1 and TNFSF13B, co‐immunoprecipitation assay was performed as previously described.^[^ ^48^ ^]^ Briefly, cell lysates were centrifuged and incubated with indicated antibodies and protein‐G bead at 4 °C overnight. Protein‐antibody complexes were eluted and then subjected to immunoblotting with corresponding antibodies. For GST pull‐down assay, GST fusion proteins and glutathione‐sepharose beads were incubated with cell lysates. Beads were subsequently harvested through centrifugation and washed four times with binding buffer and resuspended. The bound proteins were subjected to western blotting.

### Animal Experiments and Mouse Models

All mice were handled in accordance with the Guide for the Care and Use of Laboratory Animals (MX‐B4621R_20220228_100720_045). For the in vivo tumorigenesis study, stably HOOK1 overexpressed or NC‐transfected cells (1 × 10^6^ cells in 0.1 mL phosphate‐buffered saline) were subcutaneously injected into the flank of four 4‐week‐old male Balb/c nude mice, respectively. To generate a blood‐borne lung metastasis model, the Caki‐1 cells (0.5 × 10^6^) were injected into the tail vein of two groups of nude mice (8 mice/group). Eight weeks later, the mice were euthanized. The lungs were collected and metastatic nodules were counted after H&E staining. For galunisertib treatment, shRNA‐control or shRNA‐HOOK1 cells were injected into the flanks or tail vein of male nude mice, and galunisertib (800 mg kg^−1^ d^−1^) or vehicle was administered by gavage. All the mice were sacrificed at the appropriate time, and the tumors or lungs were removed for analysis.

For Tet‐on inducible sunitinib sensitivity model, four groups (four mice in each) were as follows: 1) Tet/ON‐NC group; 2) Tet/ON‐HOOK1 group; 3) Tet/ON‐TNFSF13B group; 4) Tet/ON‐HOOK1 and Tet/ON‐TNFSF13B combined group. Doxycycline diet (0.625 mg kg^−1^) was given from the second week after cells injection. The mice were orally administered sunitinib (40 mg kg^−1^ d^−1^) for a period of 4 weeks.

For sunitinib resistant xenograft mice model, the sunitinib‐resistant 786‐O cells (786‐O‐R) (1 × 10^6^) were mixed with matrigel (1:1) and injected subcutaneously into the 4‐week‐old male NSG (NOD.Cg‐Prkdc^scid^Il2rg^tm1Wjl^/SzJ) mice. The mice were orally given sunitinib (40 mg kg^−1^ d^−1^) from the second week, and randomized into four groups. The mice were intraperitoneally injected with meletin (20 mg kg^−1^) and/or subcutaneously injected with Belimumab (0.3 mg kg^−1^), while orally administered sunitinib and continued for 4 weeks.

For patient‐derived xenografts (PDX) sunitinib‐resistant model, fresh tumor samples from three RCC patients were implanted into the flank of NSG mice to generate first‐generation (P0). Sunitinib (40 mg kg^−1^ d^−1^) was started to be given at week 4 after P0 established and continued for 8 weeks. The human materials were obtained with informed consent, and the study was approved by the Clinical Research Ethics Committee (MX‐B4621R_20220210_143827_029). Then the most resistant xenograft was divided into equal pieces, and subcutaneously implanted into NSG mice as the second generation (P1). After 4 weeks, the P1 mice were divided randomly into four groups: 1) Vehicle control; 2) Meletin group (20 mg kg^−1^, twice a week); 3) Belimumab group (0.3 mg kg^−1^, twice a week); 4) Meletin and Belimumab combine group. At the end of the experiment, mice were euthanized.

For immunological model, murine RCC cell Renca (1 × 10^7^) were injected into the flank of C57BL/6 mice. When the xenografts grew to ≈50 mm^3^, mice were randomized into 4 groups and treated with anti‐PD‐1/IgG (10 mg kg^−1^, i.v.) and/or Meletin/Vehicle (20 mg kg^−1^, i.p., given at days 0, 3, 7, 10, 14). Mice were sacrificed once the tumors reached a volume of 1000 mm^3^ for tissue collection. Tumor size was measured weekly using a caliper, and the tumor volume was calculated using the following formula: *V* = 0.54 × *L* × *S* ^2^ (*V*, the tumor volume; *L*, the large diameter; *S*, the smaller diameter). Tumor weights were also recorded.

### Statistical Analysis

Statistical tests were conducted using GraphPad Prism 8.0 software. Student's *t*‐test was used for comparison between two independent groups, and variance (ANOVA) with Tukey's multiple comparisons test was used for comparison between three or more groups. Pearson correlation analysis and chi‐square test were used to examine the correlation between two continuous variables. Survival analysis was performed using the Kaplan–Meier model and two‐sided log‐rank test. Prognostic factors were assessed by univariate and multivariate Cox regression analysis. A *p*‐value of less than 0.05 was considered statistically significant.

## Conflict of Interest

The authors declare no conflict of interest.

## Author Contributions

L.Y., W.L., B.C., and H.Y. designed the study. L.Y., X.C., R.Y., and B.C. carried out in vitro experiments and collected the data. W.L., T.Z., R.W., Z.L., J.M., L.X., and H.Y. carried out in vivo experiments and collected the data. L.Y., W.L., and R.W. analyzed and interpreted the data. X.C., R.Y., and B.C. performed the statistical analysis. L.Y., W.L., X.C., and R.W. wrote the manuscript. R.Y., B.C., W.L., and H.Y. revised the manuscript. All authors have read and approved the article.

## Supporting information

Supporting Information

## Data Availability

The data that support the findings of this study are available from the corresponding author upon reasonable request.
