# Supplementary material for: HOOK1 Inhibits the Progression of Renal Cell Carcinoma via TGF‐β and TNFSF13B/VEGF‐A Axis
Source: Adv Sci (Weinh). 2023 Apr 21;10(17):2206955. doi: 10.1002/advs.202206955 (PMC10265082; doi:10.1002/advs.202206955)
Supplement: Supplementary file 1 — Supporting Information [file ADVS-10-2206955-s001.pdf]

ADVANCED SCIENCE
Open Access

Supporting Information

for *Adv. Sci.*, DOI 10.1002/advs.202206955

HOOK1 Inhibits the Progression of Renal Cell Carcinoma via TGF-β and TNFSF13B/VEGF-A Axis

Lei Yin, Wenjia Li, Xuxiao Chen, Ronghao Wang, Tao Zhang, Jialin Meng, Zhao Li, Li Xu, Rui Yin*, Bo Cheng* and Huan Yang**

Supplementary Materials

Supplementary Materials and Methods

1. Clinical specimens

Three independent cohorts of RCC surgical specimens were obtained from Ruijin Hospital and Tongji Hospital. For group 1, tissue microarrays (TMAs), 112 RCC and 30 adjacent non-tumor tissues were collected. For group 2, 4 patients suffered recurrence were enrolled, and their matched adjacent normal tissues, primary tumor, and recurrent tumor tissues were collected for further WB assay. For group 3, 4 fresh clinical samples and 4 sunitinib resistant samples were collected for WB detection. The human materials were obtained with informed consent, and the study was approved by the Clinical Research Ethics Committee.

2.Cell lines

The human normal cell line 293T, human umbilical vein endothelial cells (HUVECs), immortalized proximal tubule epithelial cell HK2, and RCC cell lines, ACHN, Caki-1, A498, OS-RC-2, Caki-2, 786-O and mouse renal adenocarcinoma cell line Renca were originally purchased from Cell Bank of the Chinese Academy of Sciences. HUVECs were cultured in EGM-2/M199. HK2 cells were cultured in Keratinocyte Serum Free Medium and the other tumor cell lines were cultured in Dulbecco's modified Eagle's medium. All cells were cultured in medium supplemented with 10% FBS in the humidified 5% CO2 environment at 37 °C. Cells were used in experiments within 6 weeks of thawing and were tested to be free of mycoplasma.

3. Antibodies and reagents

The following antibodies were used in present study: anti-HOOK1 (Abcam), anti-E-Cadherin (Cell Signaling Technology, CST), anti-N-Cadherin (CST), anti-Vimentin (CST), anti-HIF-1α (Abcam), anti-HIF-2α (Abcam), anti-VHL (Abcam), anti-E2F3 (Invitrogen Antibodies), anti-ALK5 (Abcam),

anti-phospho-Smad3 (CST), anti-phospho-MEK (CST), anti-phospho-ERK (CST), anti-c-Myc (CST), anti-VEGF-A (Abcam), anti-TNFSF13B (Abcam), anti-HA (Abcam), anti-Flag (Abcam), anti-pan-CK (Abcam) , anti-CD31 (Abcam), anti-Ki67 (Abcam), anti-PD-L1 (Proteintech), anti-PD-1 (Proteintech), anti-PDGFRB (Proteintech), anti-IL-8 (Proteintech) and anti-β-actin (Abcam). The following reagents were used in the present study: KC7F2 (Selleck), TC-S7009 (Selleck), Meletin (MedChemExpress, MCE), MG-132 (Sigma), TGF beta 1 (MCE), Galunisertib (Selleck), 10058-F4 (Selleck), Belimumab (ProteoGenix), Sunitinib (MCE), humanized anti–PD-1 antibody nivolumab (Opdivo) and human IgG (Abcam).

4. RNA extraction and quantitative real-time PCR analysis.

Total RNA was extracted using an RNeasy mini kit (Qiagen, Germany). The RNA concentration was measured using Nanodrop 2000 (Thermo Scientific, USA). cDNA was synthesized using the QuantiTect Reverse Transcription Kit (Qiagen) with the primers (Sangon Biotech, Shanghai, China) according to the manufacturer's instructions. β-actin was used to correct the difference in template input. The relative RNA expression was calculated using the 2–ΔCT method. The following primers were used in the present study: HOOK1, 5′-CAGACATTCAATACTGCCTCACC-3′ (F), 5′- CCCCAACATCCTCTTTAATTCGG -3′ (R); TNFSF13B, 5′- GGGAGCAGTCACGCCTTAC -3′ (F), 5′- GATCGGACAGAGGGGCTTT -3′(R); and β-actin, 5′- CACCATTGGCAATGAGCGGTTC -3′ (F), 5′- AGGTCTTTGCGGATGTCCACGT -3′ (R). The primers used for ChIP-qPCR are listed in:forward, 5'- CTTCCCACCCACTCTCACTC -3', and reverse, 5'- CCAGTCACAGAGACGGTAGG -3'.

5. Western blot analysis.

Briefly speaking, cells were lysed in RIPA lysis buffer containing PMSF on ice. The concentrations of proteins were determined using a BCA protein kit (Beyotime, China) and whole lysates were mixed with 6 X SDS loading buffer, heated at 100 °C for 10

min. A total of 30 mg of protein from each sample were loaded, running and then transferred to PVDF membranes. Immunoreactive bands were visualized using ECL western blot kit.

6. Migration and invasion assays.

Transwell assays were performed to evaluate migration and invasion activities. Cells were collected and washed with 1xPBS. For migration assays, 5 x 104 cells were seeded in the upper chamber with a non-coated membrane (24-well insert, pore size 8 μm; Corning, USA) with DMEM containing 1% FBS. For invasion assays, 5 x 104 cells were seeded in the upper chamber with a MatriGel-coated membrane (dilution: 1:8). In both assays, the lower chambers contained DMEM with 10% FBS as chemo-attractant. Cells were incubated at 37°C for 24 h. Cells that had migrated or invaded to the lower surface of membrane were fixed by 4% methanol, followed by staining with crystal violet. Stained cells were counted in 3 random 100X microscopic fields. All experiments were conducted in triplicate. The unpaired two-tail student's t-test was used for comparison.

7. Cell-adhesion assays.

Cell-adhesion assays were performed using the CytoSelect 48-Well Cell Adhesion Assay kit (fibronectin coated) according to manufacturer's instructions (Cell Biolabs, San Diego, CA).

8. Tube formation assay.

96-well plates and tips were incubated at -20℃ for 30 m and Matrigel (BD) was transferred onto ice. Matrigel (10 mL) was added to each well with the cool tip and distributed evenly by shaking the plate. The plate was placed on ice for 2 min and then incubated in a cell culture incubator for 30 min. Cell suspension was made by trypsinization and adjusted to 1x105 cells/mL after live cell counting. Cells were added into the pre-incubated plate at 100 mL/well, and incubated under normal conditions. Images were acquired on microscopic 6 h later. Then the enclosed

networks of complete tubes from randomly chosen fields were counted and averaged.

9. Plasmids, virus production and transfection.

To overexpress HOOK1, E2F3, HIF-1α and HIF-2α, cDNA of those genes was cloned into pWPI vector and the silence sequence was cloned into PLKO.1 backbone, respectively. The 293T packaging cells were transiently transfected with pMD2.G and psPAX2 to produce lentiviral particles. The supernatants containing lentiviral particles were collected 48 hrs post-transfection of 293T cells, filtered and used to transduce RCC cells for 48 hrs with polybrene. The viral transduced RCC cells were then subjected to 1 μg/ml puromycin selection. To generate Dox-inducible cell lines, cell lines were infected simultaneously with lentivirus encoding Tet-On 3G (packaged from pLVX-Tet3G) and lentivirus encoding TRE3G-HOOK1 or TRE3G-TNFSF13B (packaged from pLVX-TRE3G-IRES), then selected with hygromycin (200 μg/ml) and puromycin (1 μg/ml) for 2 weeks. Doxycycline hyclate (D9891; Sigma, St. Louis, MO, USA) was dissolved in ddH2O (2 mg/ml) and added to culture medium at final concentration of 2 μg/ml in order to induce overexpression of the abovementioned genes.

10. Luciferase assay

Interactions between E2FF3 and HOOK1 in RCC were verified by luciferase assay according to previous studies. Briefly, Human HOOK1 promoter region was constructed into pGL3-basic vector (Promega, USA). Then cells were seeded in 24-well plates and transfected with cDNA via Lipofectamine 3000 (Invitrogen). pRL-TK was used as negative control. The luciferase activity measured were based on the manufacturer's manual (Promega).

11. Immunohistochemistry

In general, tissue sections were incubated with primary antibodies and placed at 4°C overnight, then secondary antibodies were incubated at room temperature. IHC staining was independently assessed by three experienced pathologists. The

immunoreactive score representing the proportion of positively stained tumor cells were graded as: 0 (<10%); 1 (11%-25%); 2 (26%-50%); 3 (51%-75%) and 4 (>75%). The intensity of staining was determined as: 0 (no staining); 1 (weak staining = light yellow); 2 (moderate staining = yellow brown); and 3 (strong staining = brown). A semiquantitative scoring criterion was used, in which the staining index (values 0-12) were calculated by multiplying the staining intensity and the positive cells proportion. Finally, cases were classified into two different groups: low expression cases (score 0-6) and cases with high expression (score 7-12).

12. Multiplex immunofluorescence staining and analysis

mIHC was performed as previous described[1]. Formalin-fixed paraffin-embedded (FFPE) blocks were cut in 2 micron thick slides. The slides were baked for 60 minutes and stained using the antibodies and opal fluorophores, and nuclei were visualized with 4′,6-diamidino-2-phenylindole (DAPI). Slides were scanned using a Vectra slide scanner (PerkinElmer). For each marker, the mean fluorescent intensity per case was then determined as a base point from which positive calls could be established. For multispectral analysis, each of the individually stained sections was used to establish the spectral library of the fluorophores. Five random areas on each sample were analyzed.

13. Protein–protein docking and molecular docking

The protein crystal structure of HOOK1 and TNFSF13B was downloaded from the PDB (https://www.rcsb.org) protein database and valided in AlphaFold Protein Structure Database(https://alphafold.com). Hydrogenation, steric hindrance optimization, and hydrogen bond optimization were carried out on the initial structure to eliminate stereoscopic conflict. Protein docking was performed using HEX software. HOOK1 was selected as receptor, and the TNFSF13B structure was selected as the ligand. The docking parameters were selected based on the protein surface structure and surface potential. The 3D structures of the meletin were downloaded from PubChem (https://pubchem.ncbi.nlm.nih.gov).The HOOK1 protein structure

was prepared by assigning bond orders, by adding hydrogen, by treating disulfides, by optimizing H-bond assignment and by performing energy minimization to relax the structure using the Optimized Potentials for Liquid Simulations (OPLS)-2005 force field in a vacuum. Minimization was terminated when the root-mean-square deviation (RMSD) reached a maximum cutoff of 0.3 Å. The ligand of the crystal structure was used to define the central site of the docking grid box, and xyz dimensions of the docking grid box were set to 60 × 60 × 60. 3D.

14. Bioinformatics study

The RNA-sequencing data for ccRCC (n=533) and normal (n=72) tissue samples was downloaded from The Cancer Genome Atlas (TCGA) website (https://portal.gdc. cancer.gov/) while the corresponding clinical data of ccRCC samples was downloaded from the cBioportal website (https://www.cbioportal.org/datasets). Raw counts of RNA-sequencing data were transformed into transcripts per million (TPM) values, and were further log2- transformed (log2TPM) for subsequent analyses. Additional datasets GSE73121 and GSE76068 were downloaded from the GEO website (http://www.ncbi.nlm.nih.gov/geo/). mRNA levels derived from these datasets are arbitrarily expressed as fragments per kilobase million (FPKM). CheckMate-025 cohorts of ccRCC patients treated with Nivolumab (anti-PD-1) therapy were obtained from the published article[2]. IMvigor210 trial data (Phase 2 study investigating anti-PD-L1 in metastatic urothelial cancer, including 66 carcinoma of renal pelvis) were accessed through the IMvigor210CoreBiologies R package downloaded from http://research-pub.gene.com/IMvigor210CoreBiologies.

References

1. Goossens P, Lu C, Cao J, Gijbels MJ, Karel JMH, Wijnands E, Claes BSR, Fazzi GE, Hendriks TFE, Wouters K, Smirnov E, van Zandvoort MJM, Balluff B, et al. Integrating multiplex immunofluorescent and mass spectrometry imaging to map myeloid heterogeneity in its metabolic and cellular context. Cell Metab. 2022; 34(8):1214-1225 e1216.
2. Braun DA, Hou Y, Bakouny Z, Ficial M, Sant' Angelo M, Forman J, Ross-Macdonald P, Berger AC, Jegede OA, Elagina L, Steinharter J, Sun M, Wind-Rotolo M, et al. Interplay of somatic alterations and immune infiltration modulates response to PD-1 blockade in advanced clear cell renal cell

carcinoma. Nat Med. 2020; 26(6):909-918.

Supplementary figure legends

Supplementary Fig. 1

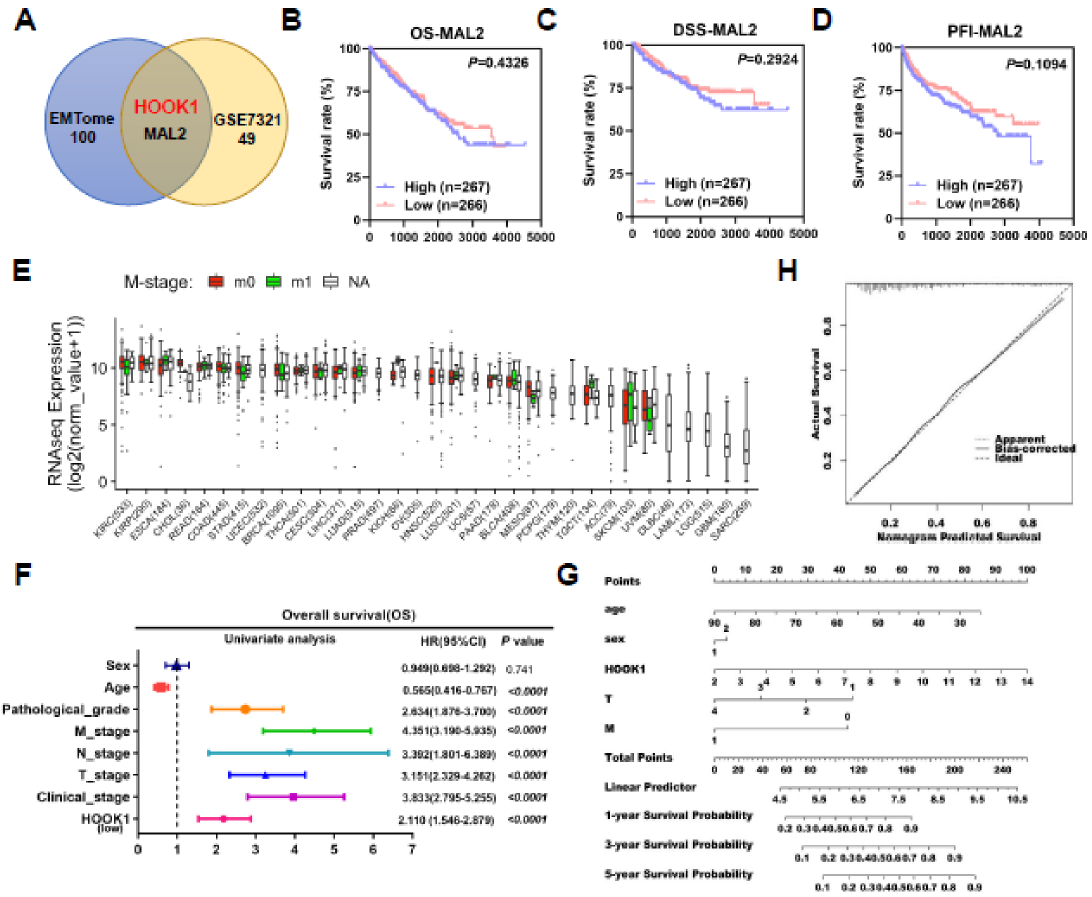

Fig. S1 Clinicopathological correlation of HOOK1 expression in RCC. **a** Venn diagram obtained from two public data: EMTome and GSE73121 dataset. **b-d** MAL2 expression was not associated with overall survival (**b**), disease-specific survival (**c**) and progression-free interval (**d**) in the TCGA-KIRC cohort according to Kaplan-Meier analysis. **e** HOOK1 mRNA expression levels in non-metastatic tumors and metastatic tissues across all TCGA pan-cancer data. **f** Forest plot showed the association between clinical parameters, HOOK1 expression and OS survival using univariate analyses. **g** Nomogram plots to predict OS at 1, 3 and 5 years after nephrectomy. **h** Validity of the predictive performance of the nomogram via calibration plots.

Supplementary Fig. 2

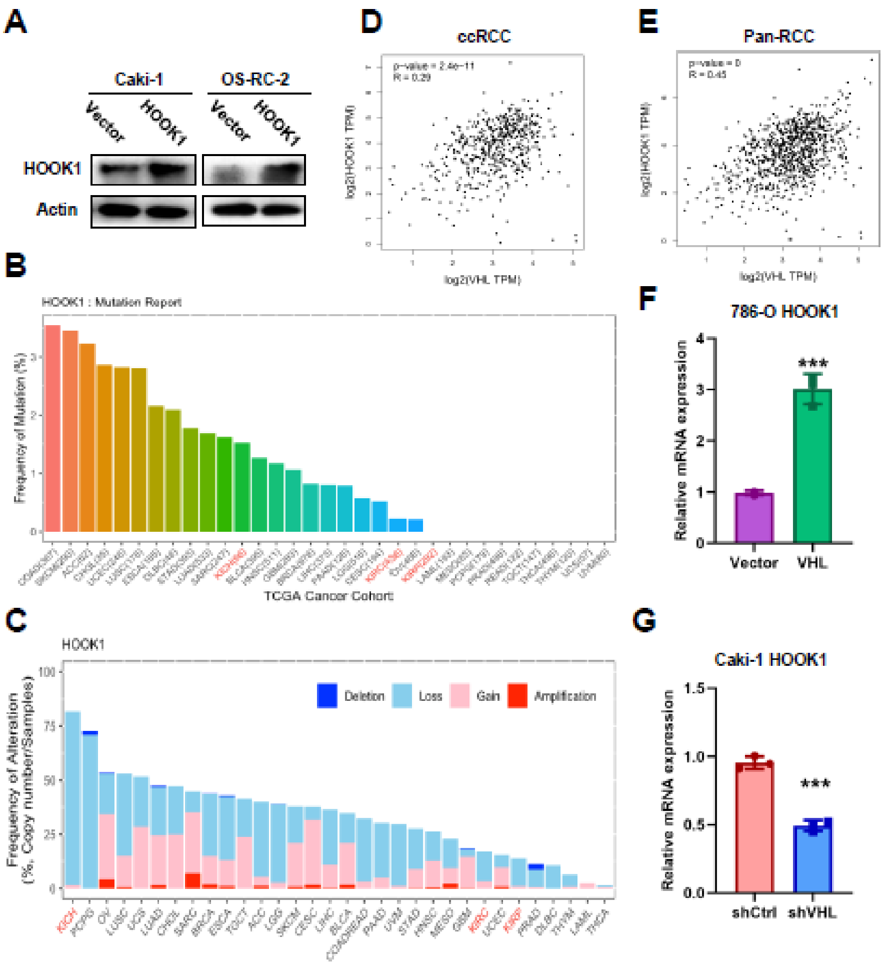

Fig. S2 The HOOK1 genetic alteration and correlation of VHL in RCC patients. **a** The overexpression of HOOK1 was detected by Western blot in Caki-1 and OS-RC-2 cells. **b** HOOK1 mutation frequency in all TCGA pan-cancer data. **c** The genetic alteration frequency of HOOK1 amplification, deletion, loss and gain in different pathological tumors. **d-e** VHL was positively associated with HOOK1 mRNA expression in TCGA-KIRC (**d**) and pan-RCC data (**e**). **f** Real-time PCR analysis of HOOK1 expression compared with control vector and wild-type VHL in 786-O cell line. **g** Real-time PCR assay for HOOK1 mRNA after knockdown of VHL in Caki-1 cell line.

Supplementary Fig. 3

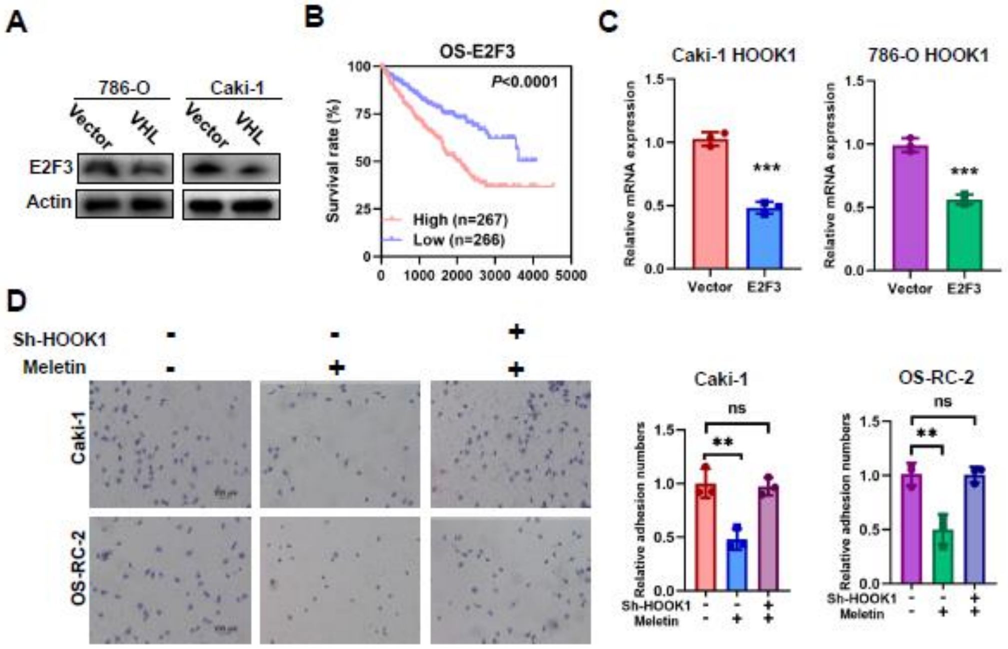

Fig. S3 Effect of E2F3 on expression of HOOK1 in renal cancer cells. **a** Immunoblotting analysis of E2F3 expression in 786-O and Caki-1 cell lines with overexpressing VHL. **b** E2F3 expression was significantly associated with worse overall survival in the TCGA-KIRC cohort according to Kaplan-Meier analysis. **c** Real-time PCR analysis of HOOK1 expression compared with control vector and wild-type E2F3 in indicated cell lines. **d** The adhesive properties of the cells were analyzed with the fibronectin adhesion assay.

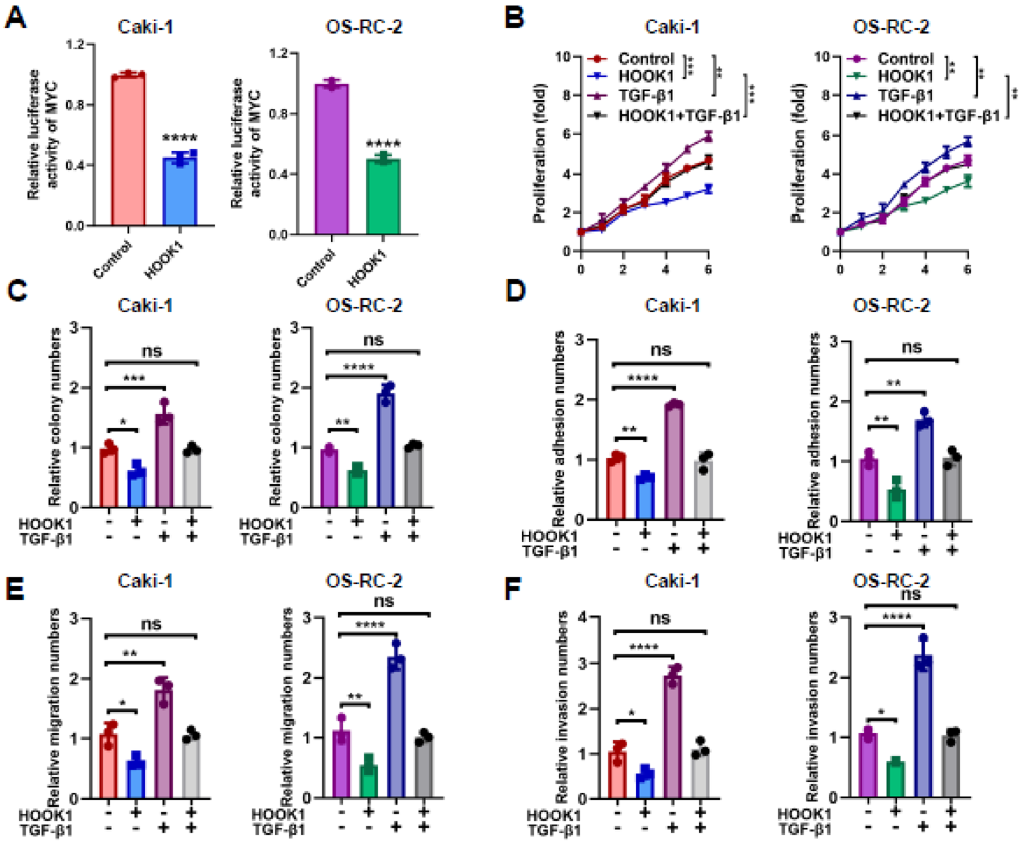

Fig. S4 TGF-β signaling is a key pathway for HOOK1 to inhibit metastasis. **a** HOOK1 regulates Myc activity in RCC cells. **b-c** Effect of TGF-β signaling on the cell viability (**b**) and colony forming ability (**c**) of HOOK1 overexpression in Caki-1 and OS-RC-2 cell lines. **d-f** Effect of TGF-β signaling on the adhesion (**d**), migration (**e**) and invasion (**f**) of HOOK1 overexpression in RCC cell lines.

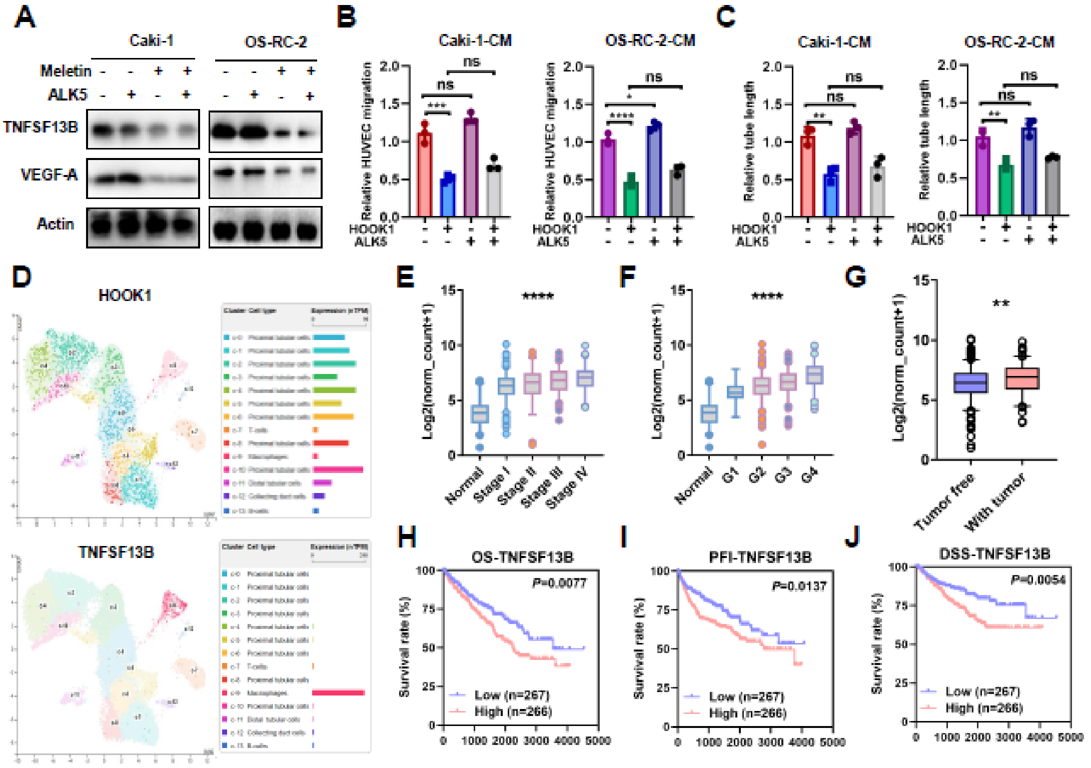

Fig. S5 TNFSF13B correlates with angiogenesis and poor prognosis of RCC patients. **a** Protein levels of TNFSF13B and VEGF-A in Caki-1 and OS-RC-2 cells under co-treatment of meletin and ALK5. **b-c** Determination of RCC-derived migration (**b**) and tube formation (**c**) of HUVECs via either HOOK1 overexpression or co-transfect ALK5. **d** UMAP plot showing the spatial location of kidney cells after dimensionality reduction. The histogram showing cluster cell type and the expression of the HOOK1 and TNFSF13B. **e-g** The association between TNFSF13B expression and clinical stage (**e**), grade (**f**) and tumor recurrence status (**g**) in TCGA-KIRC specimen. **h-j** TNFSF13B expression was significantly associated with worse overall survival (**h**), progression-free interval (**i**) and disease-specific survival (**j**) in the TCGA-KIRC cohort according to Kaplan-Meier analysis.

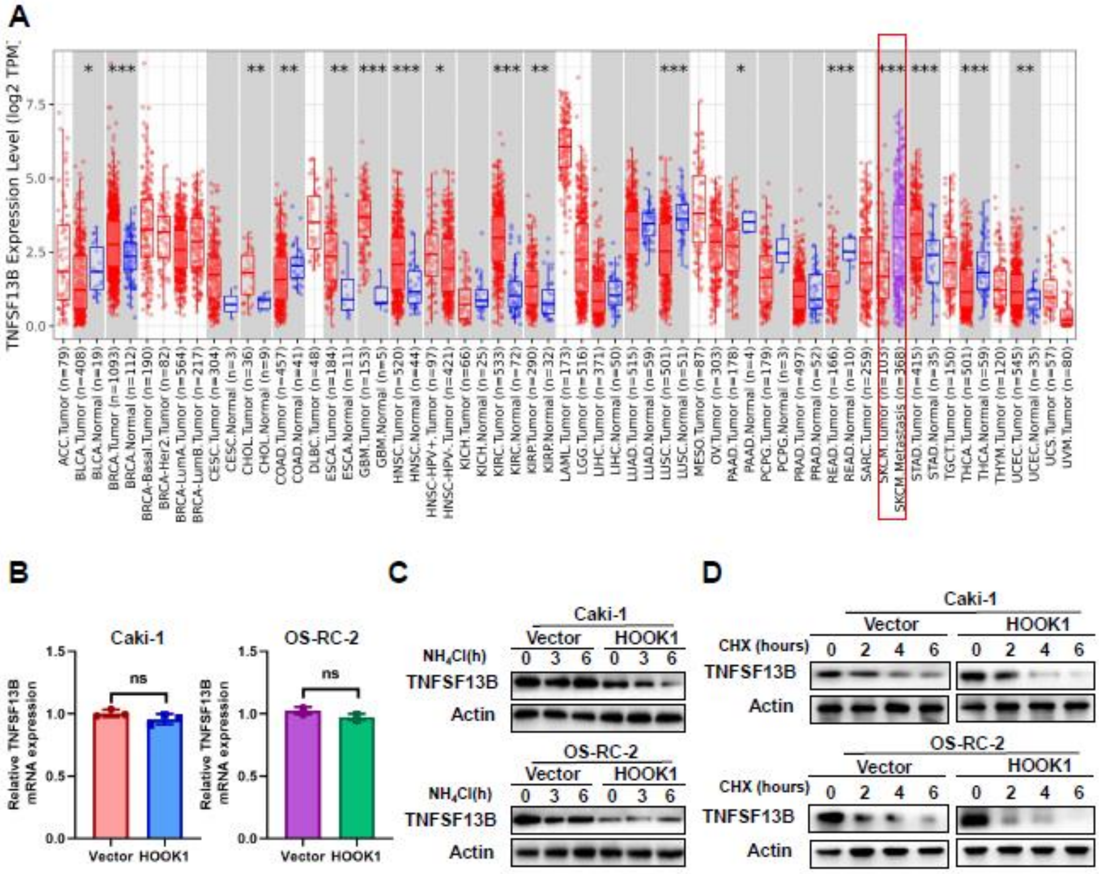

Fig. S6 HOOK1 promotes the degradation of TNFSF13B through the proteasome pathway**. a** TNFSF13B is overexpressed in multiple human tumors (TCGA database). **b** Real-time PCR analysis of TNFSF13B expression compared with control vector and HOOK1 in indicated cell lines. **c** The protein level of TNFSF13B could not rescued after autophagy lysosome inhibitor NH_4CI treatment. **d** WB analysis of TNFSF13B in RCC cells transfected with or without HOOK1 were treated with CHX at different time points.

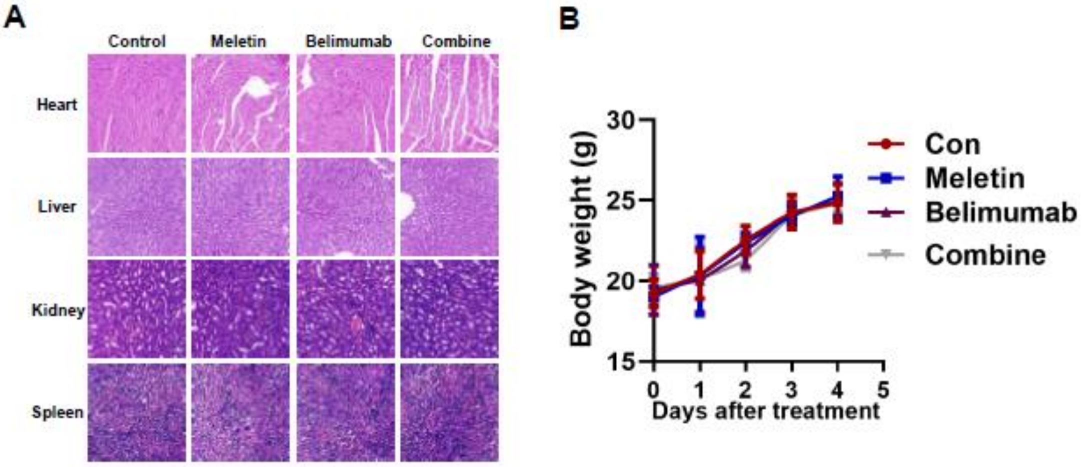

Fig. S7 No significant morphological changes in mice after Meletin/Belimumab treatment. **a** Representative images of H&E staining of heart, liver, kidney and spleen samples from Meletin/Belimumab/Vehicle treated mice. **b** Changes in murine body weights over time.

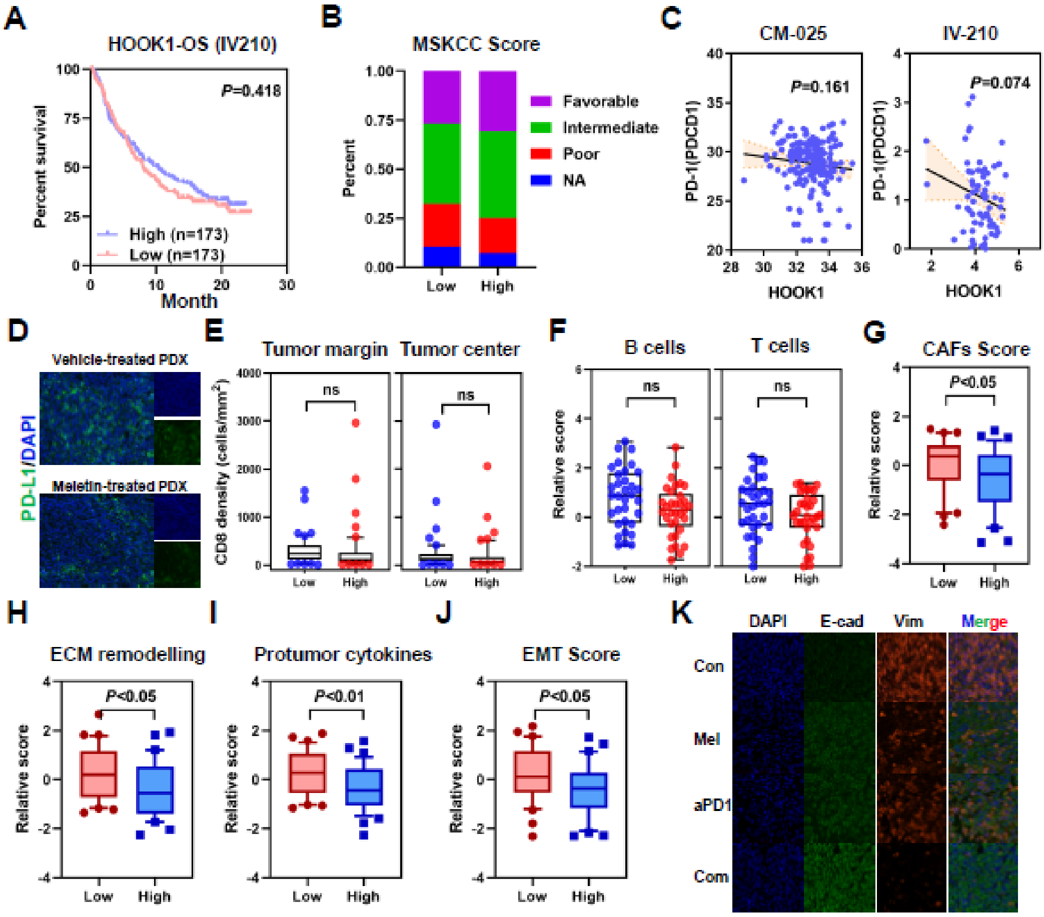

Fig. S8 HOOK1 enhances anti-PD-1 efficacy. **a** Kaplan–Meier curves showing probability of OS in HOOK1 high and HOOK1 low subgroups in IMvigor210(IV-210) phase II trial. **b** Memorial Sloan Kettering Cancer Center (MSKCC) score in CheckMate 025 (CM-025) phase III trial. **c** PD-1 expression didn't correlate with HOOK1 expression in CM-025 (left) and IV-210 (right) cohorts. **d** Representative IF staining of PD-L1 (green) in PDX samples with or without meletin treatment. **e** No association was observed between CD8 infiltration in tumor margin (left) or tumor center (right) with HOOK1 expression in CM-025 cohort. **f** No association was observed between B cells (left) or T cells infiltration (right) with HOOK1 expression in IV-210 cohort. **g-j** Low HOOK1 expression

was associated with significantly worse cancer-associated fibroblasts (CAFs) score (**g**), extracellular matrix (ECM) remodeling (**h**), protumor cytokines signature (**i**) and epithelial-mesenchymal transition (EMT) score (**j**) in CM-025. **k** Tumor sections derived from the indicated groups were stained. E-cadherin (green), Vimentin (red) and nucleus (DAPI, blue).

Supplementary Table1. Sequences for the nucleic acids used in the study.

Targets	Targeted Sequence (5'-3')
Sh-HIF-1α	CCGCTGGAGACACAATCATAT
Sh-HIF-2α	AGGTGGAGCTAACAGGACATA
Sh-HOOK1	TCATAGCCATGAGCCATATAT
Sh-VHL	TATCACACTGCCAGTGTATAC
